# From conventional to pH-sensitive formulations: increasing complexity, increasing characterization

**DOI:** 10.3389/fbioe.2026.1999697

**Published:** 2026-10-28

**Authors:** Maria Gioia Fabiano, Maria Valeria Giuli, Francesca Romana Stacchini, Luca Buccini, Daniele Passeri, Elena Del Favero, Caterina Ricci, Eleonora D’Intino, Carlotta Marianecci, Maria Carafa

**Affiliations:** 1 Department of Chemistry and Drug Technologies, Sapienza University of Rome, Rome, Italy; 2 Department of Medico-Surgical Sciences and Biotechnology, Sapienza University of Rome, Rome, Italy; 3 Department of Basic and Applied Sciences for Engineering, Sapienza University of Rome, Rome, Italy; 4 Research Center for Nanotechnology Applied to Engineering of Sapienza University of Rome (CNIS), Rome, Italy; 5 Department of Medical Biotechnology and Translational Medicine, University of Milan, Milan, Italy

**Keywords:** cancer nanotechnology, multi-technique approach, nanotechnology, pH-sensitive, stimuli-responsive

## Abstract

pH-responsive liposomal systems based on 1,2-dioleoyl-sn-glycero-3-phosphoethanolamine (DOPE) stabilized with oleic acid (OA) have been proposed as effective drug delivery platforms due to their ability to release payloads under acidic conditions. In these systems, the deprotonated form of OA (R-COO^-^) becomes protonated (RCOOH) at low pH, potentially triggering membrane destabilization. In this study, a multi-technique approach was employed to investigate the mechanisms underlying the pH sensitivity of DOPE–OA liposomes and the effect of poly(ethylene glycol) (PEG) incorporation on their behavior. Specifically, we examined (i) how OA stabilizes DOPE bilayers in a lamellar phase at physiological pH (7.4), and (ii) whether the protonation of OA under acidic conditions induces bilayer destabilization. Additionally, the liposomes were functionalized with a targeting peptide, a cyclic RGD (cRGD) peptide, to evaluate how surface modification influences key properties such as pH sensitivity, colloidal stability, and overall liposome behavior and to assess their potential for targeted drug delivery. Our findings demonstrate that OA stabilizes the DOPE bilayer by enhancing the hydrophilicity of the membrane surface. Upon acidification, the transition from RCOO^−^ to R-COOH reduces surface hydrophilicity and decreases the effective area per lipid, leading to bilayer destabilization and promoting drug release. Furthermore, PEG incorporation was found to reduce the pH sensitivity of the liposomes, in agreement with previous reports. These results provide mechanistic insights into the design of pH-sensitive liposomal systems for targeted therapeutic applications.

## 1 Introduction

The application of liposomes as drug delivery systems has been highly successful because of their ability to improve the solubility of poorly water-soluble compounds and to reduce toxicity effects of a broad variety of therapeutic agents. In addition, their high efficiency, biocompatibility, and low immunogenicity have made them valuable tools in pharmaceutical delivery (Ciriolo et al., 2025). Liposomes are composed of a phospholipid bilayer surrounding an aqueous core. Within this structure, hydrophilic substances can be enclosed in the internal aqueous compartment, whereas hydrophobic compounds can be incorporated into the lipid membrane. In recent decades, numerous strategies have been developed to prepare multifunctional liposomes for biomedical applications. Among these, microfluidic technology has emerged as a powerful alternative to conventional preparation methods due to its inherent advantages in process control and reproducibility (Pittiu et al., 2024). This approach enables precise tuning of key formulation parameters, allowing accurate control over particle size, size distribution, and other physicochemical properties, while ensuring high batch-to-batch consistency (Gharatape et al., 2025). Such control is essential for optimizing liposomal systems for applications in drug delivery, diagnostics, and vaccine development. In addition, microfluidic platforms offer scalable production, facilitating the transition from laboratory-scale formulation to large-scale manufacturing required for clinical applications (Cheng et al., 2024). The physicochemical characteristics of liposomes depend strongly on lipid composition, since the components of the bilayer influence membrane rigidity, particle size, drug release behavior, and surface charge (Chelliah et al., 2025; Menge et al., 2026). For example, liposomes formulated with saturated phospholipids, such as DMPC (1,2-Dimyristoyl-sn-glycero-3-phosphocholine), exhibit generally greater stability, slower drug release and more rigid membranes, whereas those composed of unsaturated phospholipids, such as DOPE (1,2-dioleoyl-sn-glycero-3-phosphoethanolamine), display increased membrane permeability and may offer advantages for specific drug delivery applications (Scheeder and Mela, 2025). Moreover, the incorporation of PEGylated lipids, such as DOPE-mPEG-2000, plays an important role in extending the circulation time of liposomes in the bloodstream (Ibaraki et al., 2026). Despite these advantages, conventional liposomal systems still face important limitations, including slow drug release and insufficient membrane fusion after cellular uptake into endosomal compartments. To overcome these issues, pH-responsive liposomes have emerged as a promising strategy in cancer therapy. This approach takes advantage of the fact that tumor tissues generally exhibit a more acidic environment than normal tissues, allowing liposomes to release their contents selectively at the tumor site. These pH-sensitive vesicles are engineered to remain stable under physiological conditions but become destabilized when exposed to the acidic conditions of the tumor microenvironment or at endosomal pH (at pH 4.0–5.0, the endosomal pH of tumor cells), promoting the release of the encapsulated drug (Wang et al., 2024). The acidic extracellular environment of tumors is mainly associated with increased lactic acid production from anaerobic glycolysis under hypoxic conditions and excessive carbon dioxide generation (Zhao et al., 2024). In many tumors, extracellular pH values between 5.5 and 7.0 are commonly observed. Changes in buffer pH can affect the orientation and mobility of phospholipid headgroups, promoting the migration of encapsulated molecules toward the membrane surface. This effect may result from changes in phosphate group ionization, which weaken electrostatic interactions and hydrogen bonds within the polar headgroup region. Among stimuli-responsive systems, DOPE-based pH-sensitive liposomes have been extensively investigated because their stability under physiological conditions and their ability to destabilize at acidic pH (5.5–6.5) due to protonation-induced changes in phosphatidylethanolamine (PE) headgroup interactions, with repulsive reduction (Scorzafave et al., 2025). DOPE changed the packing requirements, allowing for a packing with a smaller area per molecule at the interface of the lipid layer (Balenzano et al., 2025).

This behavior allows the liposome to remain stable in plasma and release its payload under acidic tumor conditions, improving intracellular delivery. Since DOPE alone does not readily form stable bilayers, it is usually combined with stabilizing lipids, such as phosphatidylserine, Cholesteryl Hemisuccinate (CHEMS), or Oleic Acid (OA) (Choudhury et al., 2025). Under acidic conditions, these components become protonated, promoting membrane destabilization which in turn can affect the rate of drug release (Okafor et al., 2025). In particular, at neutral pH, electrostatic repulsion between the deprotonated carboxyl groups of OA and the phosphate groups of DOPE contributes to bilayer stabilization (Nazli et al., 2024), whereas OA protonation at acidic pH promotes membrane destabilization and payload release (Sabín et al., 2025).

In this study, liposomes composed of DOPE, with or without OA, were prepared to investigate pH-sensitive behavior. Moreover, liposomes based on DMPC were formulated as a non–pH-sensitive control system. We also report the functionalization of these liposomal formulations with a synthetic cyclic RGD (cRGD) peptide (Yang et al., 2026), containing the Arginine–Glycine–Aspartate (RGD) motif, enabling high-affinity and selective binding to αvβ3 integrins. These heterodimeric transmembrane proteins mediate the interaction between the cell surface and the extracellular matrix (ECM), playing a central role in cell adhesion, migration, and survival signalling, promoting targeted drug delivery toward cells overexpressing the integrin αvβ3 receptor (Ovejero-Paredes et al., 2025). Furthermore, this work highlights the importance of applying complementary physicochemical characterization techniques to monitor the successful development of pH-sensitive liposomal formulations and to verify that their pH-responsiveness is preserved following cRGD functionalization. Such characterization is essential to confirm that the incorporation of a targeting ligand does not compromise the stimuli-responsive behavior of the nanocarrier while simultaneously providing active targeting capabilities with promising potential for future clinical applications.

## 2 Materials and methods

### 2.1 Materials

1,2-dimyristoyl-sn-glycero-3-phosphocholine (DMPC), 1,2-dioleoyl-sn-glycero-3-phosphoethanolamine (DOPE), Oleic Acid, Cholesterol (Chol), Oleic Acid, Hepes salt (N-(2-Hydroxyethyl) piperazine-N′-(2-ethanesulfonic acid) sodium salt), 1,6-diphenyl-1,3,5-hexatriene (DPH), Pyrene, Nile Red, Triton™ X-100, Human serum (from human male AB plasma, United States of America origin, sterile-filtered) and Ethanol F.U. (EtOH) were purchased by Sigma-Aldrich (St. Louis, MO, United States of America). Lissamine™ Rhodamine B (Invitrogen) and Sodium azide (NaN3) were purchased by Fisher Scientific S.R.L. DOPE-PEG2000-cRGD 18:1 (CAT#: MPL0046K) was purchased by Creative Biolabs (United States of America). Hepes buffer (pH 7.4, 10^–2^ M) was prepared by dissolving Hepes salt in distilled water and by adding NaN3 0.01% as a preservative.

### 2.2 Microfluidic setup and liposome preparation

Liposomes were prepared through Rapid Microfluidic Preparation (RMP) by using Mitos P-Pump Basic [Part No. 3200175], Bambi air compressor (model: PT-15) and Fluorinated Ethylene Propylene (FEP) Tubing, 1/16″Outside Diameter (OD) x 0.25 mm Inside Diameter (ID). Total Flow Rate (TFR, Total Flow Rate = Flow Rate of the Aqueous Phase + Flow Rate of the Lipid Solution) and the Flow Rate Ratio (FRR, Flow Rate Ratio = Flow Rate of the Aqueous Phase/Flow Rate of the Lipid Solution) were controlled using two Mitos Flow Rate Sensors (1–50 μL/min for the organic phase and 30–1,000 μL/min for the aqueous phase) and a Micromixer Chip 125 μm × 350 µm (Part #3200401, Dolomite Microfluidics, United) was used. In particular, liposome formulations were produced by mixing the lipid solution and the aqueous phase at a FRR of 20. The setup consists of two pressure pumps delivering the organic and aqueous phases through in-line valves to a T-connector and Multiflux™ interface, which connects to the microfluidic chip. The generated droplets are monitored using an integrated imaging system. In the Micromixer Chip, the lipid-containing organic solvent and aqueous buffer were injected into separate inlets and mixed rapidly through chaotic advection and diffusion within the chip’s intersecting microchannels (Figure 1). Once steady-state flow was established, each formulation was collected in glass vials and stored at 4 °C for further characterization. All liposome samples were prepared at 25 °C, which is above the phase transition temperature (T_m_) of both DMPC (∼23 °C–24 °C) and DOPE (∼−16 °C), ensuring that all lipid components were in the fluid phase during assembly. This condition facilitates proper bilayer formation and promotes uniform liposome self-assembly. Given the dilution of liposome suspensions obtained via microfluidics and to ensure complete removal of residual organic solvent, the samples were subjected to ultracentrifugation using a Beckman Coulter Optima™ MAX-XP ultracentrifuge at 30,000 *rpm* for 2 h at 10 °C. The resulting pellet was then resuspended in Hepes buffer to obtain the final formulation. The purified and concentrated samples were collected for further physicochemical characterization.

**FIGURE 1:**
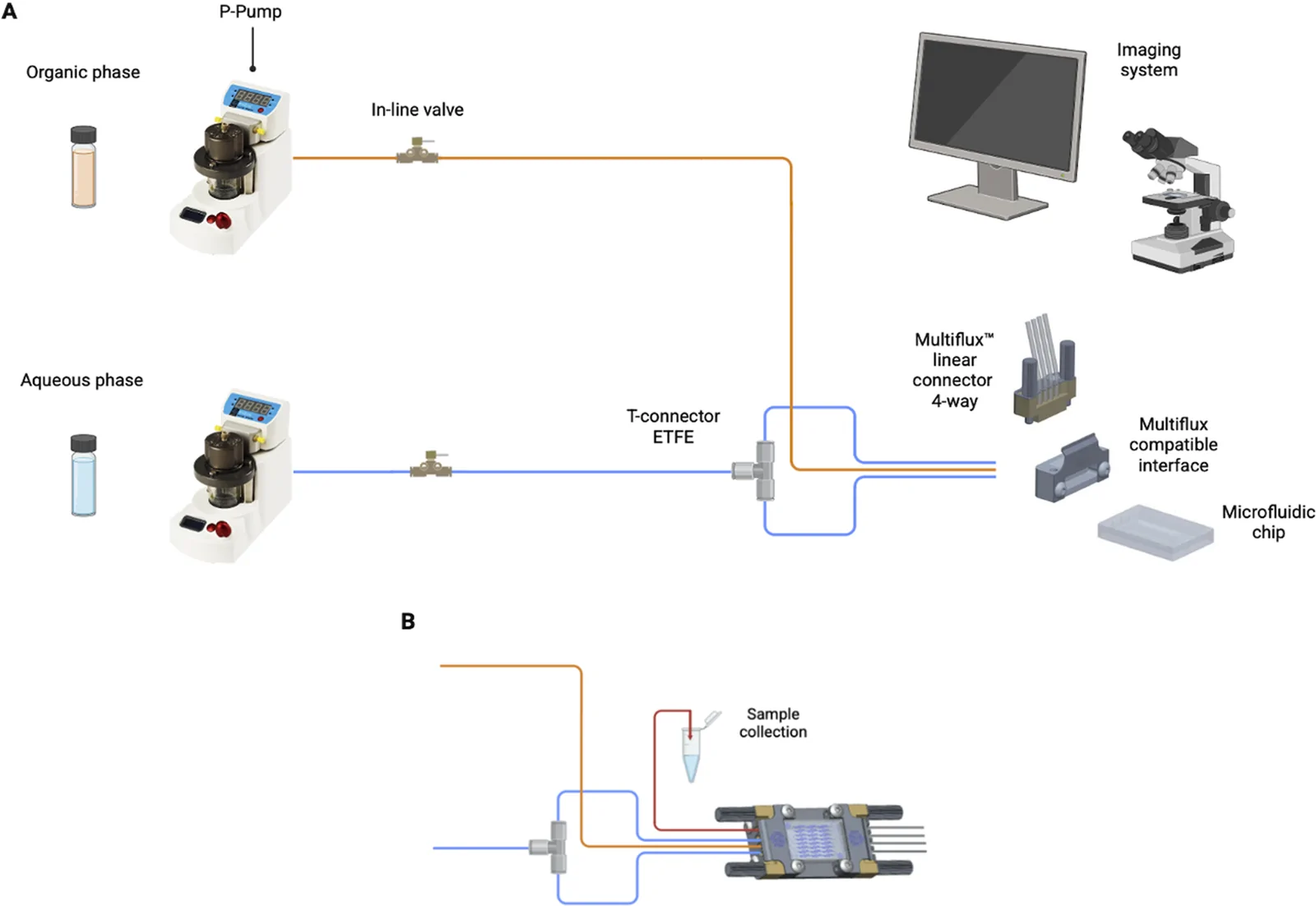
Schematic representation of the microfluidic setup used for droplet generation, including pressure pumps for the organic and aqueous phases, in-line valves and connectors **(A)**, and the microfluidic chip connected to the imaging system **(B)**. The microfluidic system scheme shows the pump infusing aqueous phase (blue) in the inputs 1 and 3 and lipid solution (orange) into the input 2, and the collection of liposomal suspension (red) obtained in the output channel. The image was created with BioRender. Adapted from CC BY 4.0, doi: https://doi.org/10.1016/j.colsurfb.2026.116203.

The main aim of this study is to prepare different DOPE-based liposomes by varying the physicochemical composition and increasing their complexity, investigating their physicochemical features. The comparison with a non-pH sensitive formulation, composed of DMPC and cholesterol, will elucidate how liposome surface composition and functionalization can affect the liposome pH-responsive behaviour. As shown in Figure 2, different formulations were exploited with increasing complexity and pH responsiveness. First, phospholipid composition is varied by adding DOPE to DMPC (1:8 mol ratio) (Figure 2A). DOPE can pack assuming an inverted cone shape more easily than DMPC, stabilizing layers with negative curvature, and promoting the spontaneous curvature of the lipid bilayer through an asymmetric distribution between the two layers. Moreover, DOPE promotes pH-sensitive behavior (Monteiro et al., 2018; Alrbyawi et al., 2022; Moreira et al., 2023). Second, a pH-sensitive helper lipid, OA, is incorporated to stabilize DOPE-containing bilayers at physiological pH while enabling destabilization under acidic conditions (Figure 2B). Third, surface functionalization on samples A and B is introduced by decorating the liposome with targeting moieties (PEG2000-cRGD) (Figure 2C).

**FIGURE 2:**
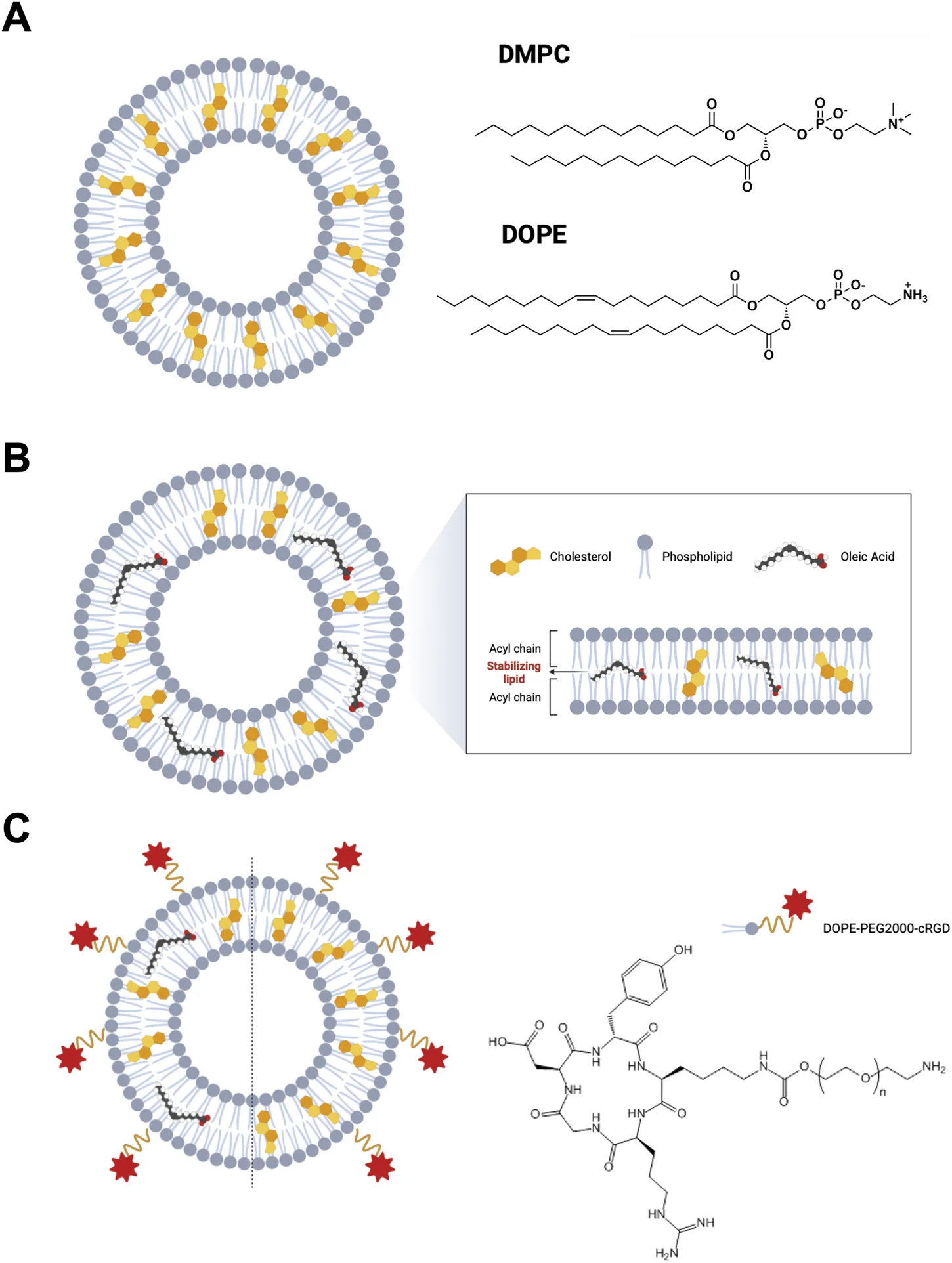
Schematic representation of the three liposome design strategies. **(A)** Conventional liposomes composed of DMPC, DOPE, and cholesterol. **(B)** Liposomes with the same basic composition as in panel A, incorporating the stabilizing lipid OA, shown as distinct molecules interspersed within the lipid bilayer. **(C)** Surface-functionalized liposomes derived from both formulations **(A,B)**, decorated with PEG chains and targeting ligands. The inset provides an enlarged view of the chemical structure of a representative targeting ligand, c(RGDyK). The image was created with BioRender.

Moreover, samples composition is reported in Table 1.

**TABLE 1:** Samples composition.

Sample	DMPC (mM)	Chol (mM)	DOPE (mM)	OA (mM)	DOPE-PEG2000-cRGD 18:1 (mM)
Lipo-DMPC	8	4	-	-	-
Lipo-DOPE	8	4	1	-	-
Lipo-OA	8	4	1	5	-
Lipo-cRGD	8	4	-	-	1
Lipo-OA-cRGD	8	4	-	5	1

### 2.3 Dynamic light-scattering (DLS) measurements: determination of particle size and zeta potential of liposomal formulations

To ensure liposomal formation and characterize all the formulations in terms of hydrodynamic diameter, PDI and ζ-potential, the samples are analysed using a Malvern NanoZetaSizer apparatus (Zetasizer PRO ZSU 58000, Malvern Panalytical, United States) with a scattering angle of 173° and 13° and equipped with a 4 mW HeNe laser (λ = 632.8 nm) (D’Intino et al., 2025). The vesicle dispersions were measured at 25 °C directly without dilution, except for the pH sensitivity studies, where dilution was necessary because the samples were brought into contact with different buffer solutions and compared at the same dilution.

### 2.4 Structural characterization

To evaluate the influence of liposomal composition on their overall properties, a series of structural characterization analyses were performed, including Small Angle X-ray Scattering (SAXS), Atomic Force Microscopy (AFM) and pH-sensitivity evaluation assays. These complementary techniques provided detailed information on size distribution, bilayer features and internal organization.

#### 2.4.1 Small Angle X-ray scattering (SAXS)

SAXS experiments were performed at the ID02 beamline of the European Synchrotron Radiation Facility (ESRF, Grenoble, France (Ricci and Del Favero, 2026; Narayanan et al., 2022). Sample and buffers were inserted in plastic capillaries (ENKI, Concesio, Italy) mounted in a multi-place sample holder and measured in identical conditions at 25 °C. The scattered intensity was acquired at two sample-to-detector distances (1 and 10 m), corresponding to a scattering vector (q) range 0.01–6 nm^-1^. The scattering vector is defined as q = (4π/λ) sin(θ/2), where λ (0.1 nm^-1^) is the X-ray wavelength and θ is the scattering angle. After correction for background and normalization, the 2D patterns were azimuthally averaged to obtain the scattered intensity profiles I(q). The structural evolution of formulations under acidic pH were assessed by comparing SAXS scattered intensity profiles at pH 7.4 and 5.5 (Barattin et al., 2018). The intensity profiles were analysed using SasView software (version 6.0.0), using a random lamellar head/tail/tail/head sheet with Caillé structure factor model, suitable for the description of stacked lipid bilayers. The overall membrane thickness, the lamellar repeat distance, the number of correlated layers, and bilayer fluctuations through the Caillé parameter could be obtained. For fitting purposes, the scattering length densities of the lipid tails, headgroups, and solvent were fixed to physically realistic values, while the lamellar repeat distance (*d*-spacing), number of correlated layers (*N* layers), and Caillé parameter (η) were refined. The fitted parameters were used to compare the degree of lamellar ordering among the different liposomal formulations.

#### 2.4.2 Atomic force microscopy (AFM)

The morphological analysis of the samples was performed using atomic force microscopy (AFM). A Cypher VRS microscope (Asylum Research) was operated in contact mode using DNP probes (Bruker Inc.). The samples were deposited onto silicon substrates, and images were acquired at room temperature after solvent evaporation. The acquired images were subsequently post-processed using Gwyddion software (version 2.62) to remove background and perform leveling.

### 2.5 pH-sensitivity evaluation

A detailed physicochemical characterization of liposomal formulations is critical to fully elucidate their different pH-sensitive behavior as it provides essential information on key parameters such as vesicle size, bilayer organization, and fluidity depending on external pH. Understanding these properties allows for the assessment of liposome stability under different environmental conditions and offers insights into how surface modifications, such as cRGD functionalization and PEGylation, influence their structural integrity and responsiveness to acidic pH. Moreover, such comprehensive analyses are fundamental for predicting the performance of liposomes as drug delivery systems, including their potential for controlled release, cellular uptake, and targeting efficiency, thereby guiding the rational design of pH-responsive nanocarriers for therapeutic applications.

To evaluate the effect of pH on the average vesicle size and to assess pH-induced liposomal aggregation, pH-sensitive liposomes were compared with non-pH-sensitive formulations. The following media were used: acetate buffers at pH 4.4 and 5.5 (to mimic when endosomes mature into lysosomes and late endosomes pH, respectively), and Hepes buffers at pH 6.8 (early endosomes) and 7.4 (physiological pH) (Saraf and Jain, 2023).

Liposomal samples were diluted in the different buffers and incubated at 37 °C ± 1 °C. At different time points, samples were taken and analysed for vesicle size using a Malvern NanoZetaSizer apparatus (Zetasizer PRO ZSU 58000, Malvern Panalytical, UK).

Turbidity measurement is a convenient assay to check the changes of particle size and one of the easy methods to evaluate the effect of aggregation and structure changes of liposomes. Turbidity of liposomal dispersion as a function of pH was followed by spectrofluorimetric monitoring at λex, λem: 600 nm (Yang et al., 2026).

To evaluate how pH could affect liposomal bilayer fluidity, the lipophilic fluorescent probe 1,6-diphenyl-1,3,5-hexatriene (DPH) was added to vesicle composition. DPH is a widely used fluorescent probe for assessing the anisotropy—and thus the fluidity—of lipophilic membranes. The probe preferentially locates within the hydrophobic core of lipid bilayers, making it suitable for evaluating this region and the effects of pH variation. Within the membrane, DPH molecules tend to align parallel to the lipid chains. The measured anisotropy depends on the rotational mobility of the probe within the bilayer and is highly sensitive to the packing order of lipid chains and overall membrane fluidity. DPH-labeled liposomes were prepared as reported above, with the additional step of dissolving 33 μL/mL of a DPH stock solution (2 mM, prepared in dichloromethane, DCM) into the organic phase to give a final probe-to-lipid ratio of 1:200 (Silva et al., 2007; Stott et al., 2008). To allow the fluorescent probe disposition in the liposomal bilayer, samples were stored 3 h at room temperature before the fluorescence anisotropy experiments. Anisotropy measurements were carried out with a Perkin-Elmer LS50B spectrofluorimeter at excitation (λex) and emission (λem) wavelengths of 350 nm and 428 nm, respectively. Fluorescence anisotropy (A) was calculated using the following formula:

$$A=\frac{\left(Ivv-Ivh\right)\cdot G}{\left(Ivv+2\cdot Ivh\right)\cdot G}$$

where I_VV_, I_VH_, I_HV_ and I_HH_ represent the fluorescence intensities measured with vertical (V) and horizontal (H) orientations of the excitation and emission polarizers. The G factor is defined as G = I_HV_/I_HH_, accounting for differences in detector sensitivity. Anisotropy reflects the rotational freedom of the probe, providing information about membrane fluidity, lipid packing, and interactions with incorporated molecules. Moreover, bilayer polarity and microviscosity were determined by fluorescence experiments on liposomes loaded with 4 mM of pyrene, used to detect the lateral distribution and mobility of the enveloping membrane compounds. The fluorescence experiments on liposomes incorporating pyrene were carried out to evaluate the micropolarity of the lipophilic bilayer by a Perkin-Elmer LS55 spectrofluorometer. Emission spectra were recorded from 350 to 550 nm using an λex: 330 nm. Polarity was estimated using the vibrational peak intensity I_1_/I_3_ ratio, while microviscosity using the I_E_/I_3_ ratio. All measurements were done 30 min after mixing of liposomes and buffers.

To further assess the effect of cRGD functionalization and PEGylation on pH sensitivity, Lissamine™ Rhodamine B was incorporated into the selected liposomal samples. Rhodamine dyes are highly sensitive to pH and were used to monitor changes in liposome behavior under different acidic conditions (Tian et al., 2012; Pandey et al., 2025). The rhodamine moiety, excitable at λex: 315 nm and at λex: 560 nm in its ring-opened form, acts as a pH-responsive fluorophore. In this context, rhodamine derivative was used as a fluorescent probe due to its excellent photophysical properties, including long absorption and emission wavelengths in the visible region, high fluorescence quantum yield, and large absorption coefficients (Hu et al., 2013; Zhang et al., 2025). Fluorescence spectra were acquired using a Perkin-Elmer LS50B spectrofluorimeter, with liposomal samples diluted 1:10 in the corresponding buffer prior to measurement. To determine the total amount of rhodamine encapsulated, liposomes were completely disrupted by the addition of 0.5% Triton X-100, providing a control for the maximum entrapped probe.

This setup allowed for the quantitative monitoring of pH-dependent structural changes and the corresponding release of rhodamine from cRGD-functionalized and PEGylated liposomes under different acidic conditions. The release of the fluorescent probes from liposomal formulations was evaluated using the dialysis method. Hepes buffer (pH 7.4) Acetate buffer (pH 5.5 and 4.4) and HS, each placed in 15 mL Falcon™ conical tubes, were used as receptor phases. SpectraPor® regenerated cellulose dialysis tubing with a molecular weight cut-off (MWCO) of 8 kDa, pre-wetted in 0.05% sodium azide, was employed. One milliliter (mL) of the liposomal formulation was loaded into the dialysis tubing, which was then immersed in the receptor phase. The encapsulated probes were present at a self-quenching concentration inside the liposomes, resulting in low fluorescence intensity; upon release and subsequent dilution in the surrounding medium, fluorescence was restored, enabling quantitative detection. All samples were incubated at 37 °C for 72 h in a rotary shaker operating at 150 *rpm*. At predetermined time intervals, 1 mL samples were withdrawn from the receptor phase to be analysed and immediately replaced after each after analysis. Fluorescence measurements were performed using a PerkinElmer LS50B spectrofluorometer. Calcein was analysed at an excitation wavelength of 492 nm and an emission wavelength of 520 nm, while FITC (fluorescein-5-isothiocyanate) was measured at 498 nm (excitation) and 517 nm (emission). Calibration curves were previously constructed for both probes in Hepes buffer (pH 7.4) and Acetate buffer (pH 5.5 and 4.4) to ensure accurate quantification of the released fluorescence signal. Calcein and FITC are fluorescein derivatives and their emissive properties are governed by the same acid–base equilibria of the xanthene core. Intensity values were normalized to a reference condition at near-neutral pH, where the probe is predominantly in its highly fluorescent, deprotonated form, analogous to the dianionic species of fluorescein. Under more acidic conditions, where protonation of phenolic and carboxyl groups occurs, a systematic decrease in fluorescence intensity was observed and incorporated into data analysis by applying a pH calibration curve. To determine the total amount of encapsulated probe, liposomes were completely disrupted by the addition of 0.5% Triton X-100, allowing the release of all entrapped fluorophores. The cumulative percentage of probe release (%) was plotted as a function of time. All experiments were conducted in triplicate, and the results are presented as mean values ± standard deviation (SD) of the mean.

### 2.6 Stability studies

Stability over time of the suspensions was measured by repeated analyses of their hydrodynamic diameter (H_D_), PDI and ζ-potential at room temperature (RT) and 4 °C for 90 days. Each sample was analysed at different times after preparation (daily for the first week, 14, 21, 30, 60 and 90 days). Measurements were performed on at least three different preparations for each sample and results were reported as mean values ± SD of the mean.

For biological stability assessment, the formulations were characterized by repeated measurements of H_D_ and ζ-potential using DLS. Samples were maintained under magnetic stirring in a 45% serum media at 37 °C for 72 h to simulate *in vitro/in vivo* conditions, providing information on colloidal stability in HS over time. In complex biological environments containing salts, proteins, lipids, and other biomolecules, liposomes are exposed to multiple interactions that can alter their physicochemical properties and ultimately affect their stability and behavior. DLS measurements were performed at different time points after the beginning of the experiment (5, 10, 15, and 30 min, data not shown), then hourly during the first 8 h, and subsequently at 24, 48, and 72 h. After measurement, each aliquot was reintroduced into the acceptor medium.

Calcein/FITC *in vitro* release studies in HS at 37 °C for 72 h were performed to assess whether the formulations behave pH-sensitivity under the selected conditions, and to assess whether the formulations remain stable in HS and behave appropriately under physiologically relevant conditions. They were carried out by sampling every hour for the first 8 h and then at 24, 48 and 72 h. 1 mL aliquots of the release medium were sampled every hour. The fluorescence of each aliquot was measured immediately by spectrofluorimeter as described above. After measurement, each aliquot was reintroduced into the acceptor medium to preserve sink conditions and maintain a constant volume throughout the experiment.

### 2.7 Statistical analyses

Statistical analyses were performed using GraphPad Prism 10 for macOS, Version 10.6.1 (GraphPad Software, San Diego, CA, United States of America). The experiments conducted to optimize the liposomal formulation and production protocol were carried out with at least three independent replicates. The results are shown as mean ± SD of the mean. One-way analysis of variance (ANOVA) was carried out to compare the size and PDI between groups when different parameters were varied. A two-way ANOVA was also performed to assess the combined effects of two independent variables on the measured parameters, followed by Tuckey’s multiple comparisons test or by Sidak’s multiple comparison tests. Results were considered statistically significant at (p < 0.05, *), (p < 0.01, **), (p < 0.005, ***) and (p < 0.0001, ****).

## 3 Results and discussion

### 3.1 Liposome production and downstream processing

Liposomes were successfully produced using the Micromixer Chip. Following microfluidic production, liposome suspensions were subjected to downstream processing to remove residual organic solvent and unincorporated components. Moreover, they were resuspended by accounting for the dilution factor resulting from the microfluidic mixing process.

SAXS measurements were initially performed on Lipo-DOPE, the base formulation for all pH-sensitive systems, prepared at different FRRs to investigate whether the microfluidic operating conditions affected the internal organization of the liposomes. SAXS scattering profiles are reported in Figure 3.

**FIGURE 3:**
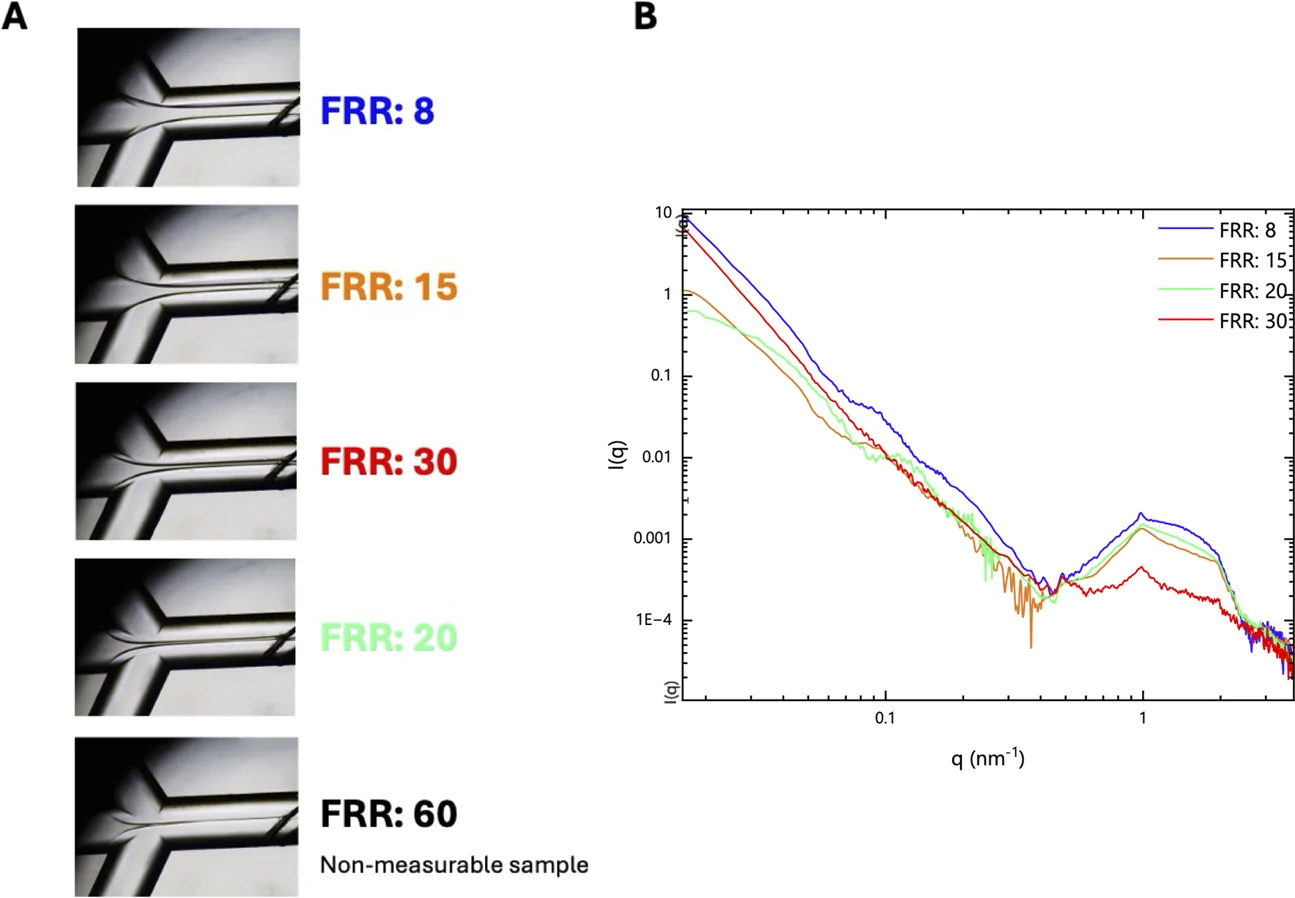
SAXS analysis of Lipo-DOPE formulations prepared at different flow rate ratios (FRRs). Panel **(A)** shows representative optical micrographs of the focused stream within the microfluidic chip at FRR values of 8, 15, 20, 30, and 60. Panel **(B)** reports the corresponding SAXS scattering profiles as a function of the scattering vector q.

All formulations exhibited the characteristic scattering signature of vesicular lipid assemblies, confirming successful liposome formation under all investigated conditions. Differences among the scattering curves were observed in the low-q region, where the scattering intensity is sensitive to the overall particle dimensions. Samples prepared at low (8) or high (30) FRRs displayed higher scattering intensity at low q values, reflecting the presence of larger vesicular structures. At 15 and 20 FRR the intensity plateau and the minimum position at 0.1 nm^-1^ indicated a decrease in vesicle size as a consequence of faster solvent exchange and more rapid lipid self-assembly.

In the intermediate-q region, all formulations exhibited a correlation peak centered around q ≈ 1 nm^-1^, which is characteristic of a spherical lipidic shell organized in a multi-bilayer arrangement. The inter-bilayer distance remained substantially unchanged across the investigated FRRs (about 6 nm), suggesting that the different vesicles were partially multilamellar, with similar internal bilayer organization. At 30 FFR the vesicle shape factor, with the characteristic bump in the region 0.5–2.5 nm^-1^, became less visible, suppressed by a high multilamellarity component. SAXS results suggest that very high FRR values are not necessarily associated with improved structural organization.

Overall, these findings suggested that an FRR of 20 provided the best compromise between size, monodispersity and unilamellarity. In particular, for all following analyses and sample preparations, a flow rate of 800 μL/min for the aqueous phase and 40 μL/min for the organic phase was employed. The obtained liposomes and their physicochemical features are reported in Table 2.

**TABLE 2:** H_D_, PDI and ζ-Potential values of the liposomal formulations.

Sample	H_D_ (nm) ± SD	PDI ± SD	ζ-Potential (mV) ± SD
Lipo-DMPC	210 ± 2	0.23 ± 0.03	−9.9 ± 0.1
Lipo-DOPE	156 ± 3	0.19 ± 0.05	−30.0 ± 0.1
Lipo-OA	89 ± 1	0.14 ± 0.05	−42.7 ± 0.1
Lipo-cRGD	198 ± 2	0.22 ± 0.03	−25.2 ± 0.1
Lipo-OA-cRGD	108 ± 2	0.19 ± 0.03	−24.3 ± 0.1

As shown, all samples exhibited hydrodynamic diameters in the range of approximately 90–210 nm, which is within the typical size range for liposomal formulations suitable for cellular uptake. The PDI values were all below 0.25, indicating relatively narrow size distributions and homogeneous populations. The ζ-potential values varied depending on the lipid composition and surface modification. Lipo-DOPE showed the highest negative charge (−42.7 mV), suggesting good electrostatic stabilization, whereas Lipo-DMPC exhibited a lower negative charge (−9.9 mV). Surface functionalization with cRGD slightly reduced the ζ-potential of the liposomes compared to their unmodified counterparts, consistent with the incorporation of peptide moieties at the liposomal surface.

### 3.2 Structural characterization: atomic force microscopy and small angle X-ray scattering (SAXS)

AFM analysis was performed to study the morphological characteristics of the five different liposomal formulations after deposition on silicon substrates and solvent evaporation. All observed particles exhibited dispersed nanometric structures, confirming the typical spherical morphology of liposomes in all samples, as observed in Figure 4. The diameters measured from the AFM images were 63 ± 18 nm for Lipo-DMPC, 52 ± 10 nm for Lipo-DOPE, 58 ± 14 nm for Lipo-OA, 57 ± 11 nm for Lipo-cRGD, and 51 ± 12 nm for Lipo-OA-cRGD. These values are lower than the corresponding hydrodynamic diameters obtained by DLS. In fact, when analysed in the dry state, this type of system may exhibit flattening of the particles due to adsorption onto the substrate, dehydration, and the stiffness of the probe. Overall, the AFM results confirm the presence of nanometric structures in all the formulations analysed.

**FIGURE 4:**
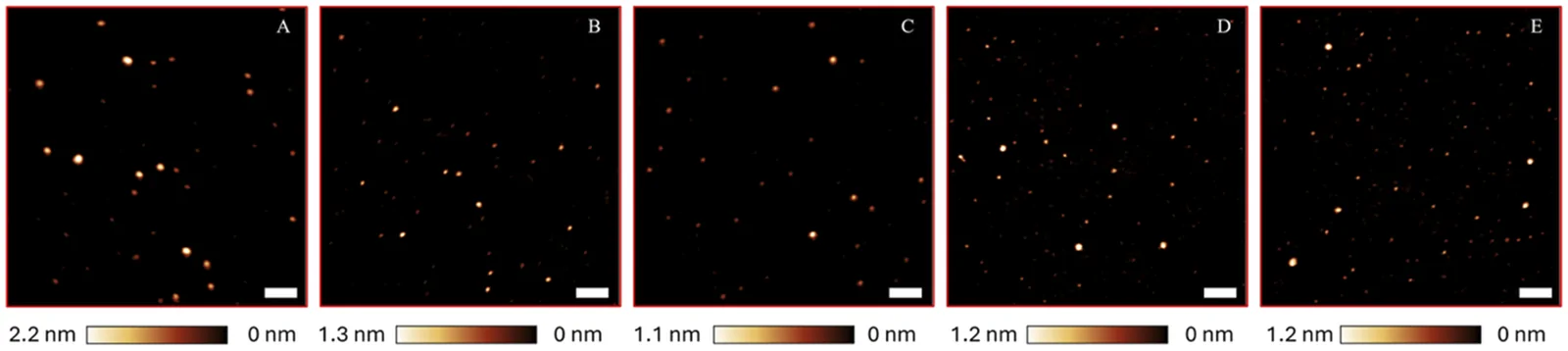
Atomic Force Microscopy analysis performed on samples Lipo-DMPC **(A)**, Lipo-DOPE **(B)**, Lipo-OA **(C)**, Lipo-cRGD **(D)** and Lipo-OA-cRGD **(E)**.

The structural properties of liposomes were assessed by SAXS. All spectra, reported in Figure 5, show intensity profiles characteristic of vesicular assemblies, displaying a first minimum whose position is related to their overall size. The addition of DOPE and OA affected the size of DMPC based liposomes. The size reduced from about 200 nm to less than 100 nm, in agreement with DLS analysis. Also, the degree of multilamellarity is modulated, being Lipo-DMPC > Lipo-DOPE > Lipo-OA. The additional surface functionalization with cRGD affected mostly the multilamellarity. The intensity bumps in the region 0.5–2.5 nm^-1^, characteristic of the lipid bilayer, are well visible, together with well-defined peaks at q = 1 nm^-1^. The results suggest the presence of a small, well organized, fraction of stacked lipid bilayer bilayers. Overall, the obtained data indicate that the liposomes are of suitable size, exhibit low polydispersity, and possess surface charges conducive to colloidal stability, with minor modulation by lipid composition and surface functionalization.

**FIGURE 5:**
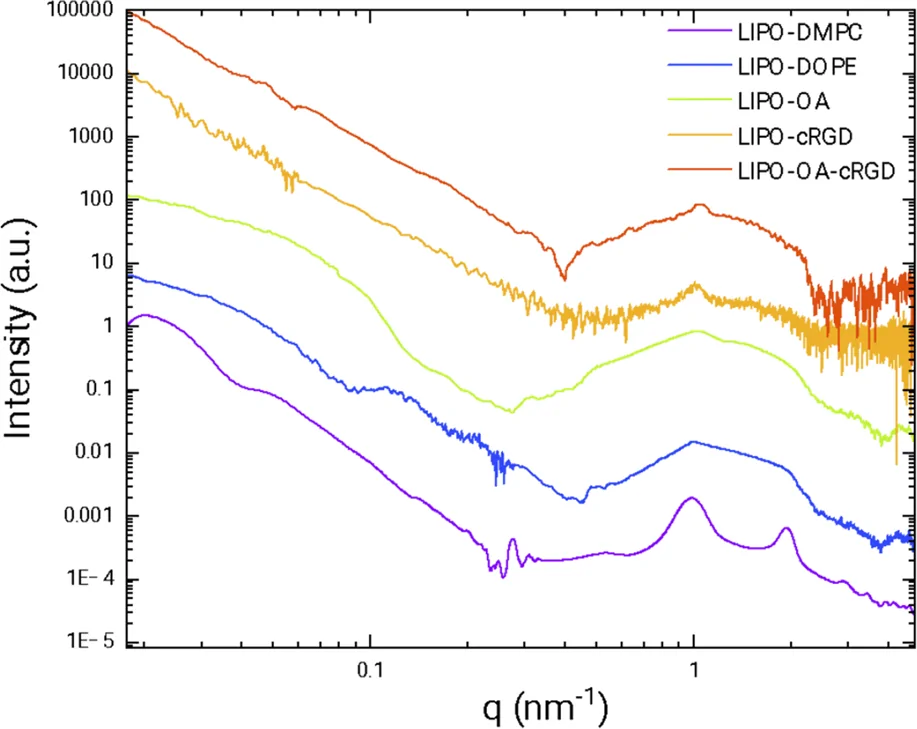
SAXS intensity profiles of liposomal formulations prepared at FRR: 20.

### 3.3 pH-sensitivity evaluation

The pH-dependent behavior of liposomes was determined by measuring the hydrodynamic diameter changes as a function of buffer pH. As shown in Figure 6, Lipo-DMPC, as non-pH-sensitive, displayed the same vesicle size and no significant variation upon decreasing the pH from 7.4 to 4.4, indicating the absence of pH-triggered destabilization. In contrast, all the other formulations exhibited a marked increase in hydrodynamic diameter as the pH decreased, particularly at pH 5.5 and 4.4. This size increase was irreversible, suggesting fusion or aggregation phenomena (Gnopo et al., 2020; Shi et al., 2022). The structural evolution of liposomal formulations at acidic pH values was observed by SAXS (see Figure 7). Data were analysed using a random lamellar head/tail/tail/head sheet and, in presence of a high degree of multilamellarity, with a Caillé structure factor model, which describes stacked lipid bilayers including membrane thickness, lamellar repeat distance, and bilayer fluctuations through the Caillé parameter. Detailed results are reported in the Supporting Information. At pH 5.5 the thickness of the headgroup layers slightly decreased (0.2 nm) in Lipo-DMPC, Lipo-DOPE, and Lipo-OA systems, while major differences are visible in the order of multilamellar bilayers. Lipo-DMPC was already highly multilamellar at pH 7.4, displaying a highly ordered lamellar arrangement and strong interlamellar correlation (Caillé parameter η ≈ 0.1) and did not show any remarkable change at lower pH (5.5), as expected. Differently, the structure of Lipo-DOPE and Lipo-OA formulations underwent a significant evolution upon pH lowering. At pH 5.5 both formulations increased multilamellarity, with a Caillé parameter η ≈ 0.3 for Lipo-DOPE and η ≈ 0.25 for Lipo-OA, indicating moderately flexible membrane stacks. These changes are consistent with a loss of lamellar order, increased bilayer fluctuations and rearrangement of the lamellar organization. In the presence of PEG2000-cRGD the effect on the internal rearrangement of the lamellar stack was mitigated by surface functionalization, suggesting a role of PEG in the maintenance of liposome integrity. The overall behavior at acidic pH values can be attributed to the protonation of pH-sensitive lipid components, such as OA, which reduces electrostatic repulsion and leads to a gradual destabilization and rearrangement of the lamellar organization. The enhanced pH-dependent interaction under mildly acidic conditions, mimicking endosomal environments, highlights the potential of these formulations to undergo structural transitions that may facilitate intracellular delivery. Furthermore, the absence of this effect in Lipo-DMPC confirms its role as a non-pH-responsive control, supporting the contribution of lipid composition to the observed behavior. SAXS data indicate that acidification mainly affects the degree of lamellar ordering and the correlation among stacked bilayers, leading to a progressive structural reorganization of the lipid assemblies. Moreover, the structural evolution of liposomal formulations under acidic pH were assessed by comparing SAXS scattered intensity profiles at pH 7.4 and 5.5 (See Supplementary Figures 1, 2; Supplementary Tables 1, 2)*.* SAXS analyses were also carried out at pH 4.4 and they confirmed the behavior of pH-sensitive samples, more evident for OA samples, but results are not reported here for brevity.

**FIGURE 6:**
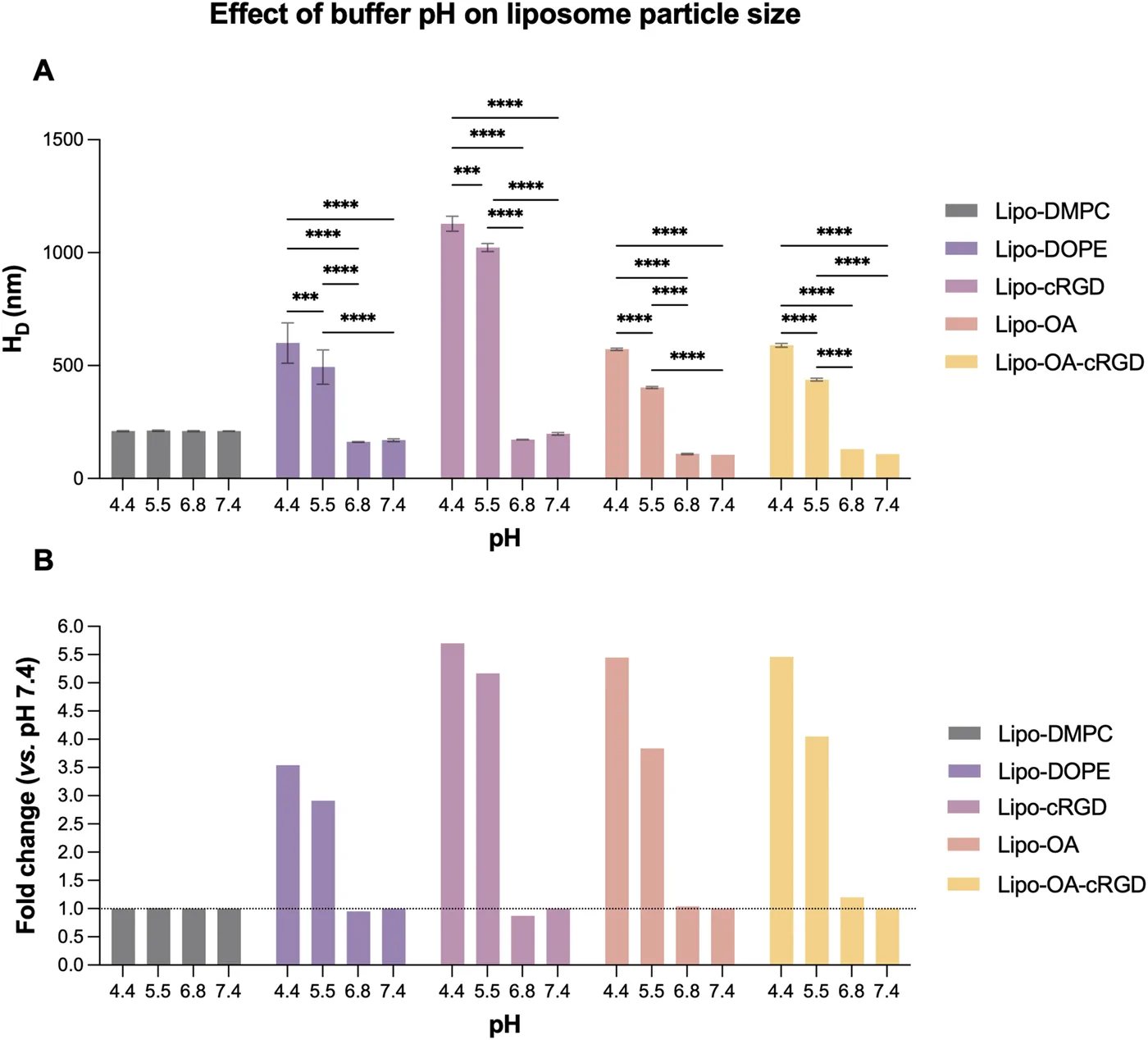
pH-dependent change in liposome particle size **(A)** and fold change **(B)** after incubation of pH-sensitive (Lipo-DOPE, Lipo-OA and the cRGD-functionalized counterparts) and non-pH-sensitive (Lipo-DMPC) samples. Fold change was calculated relative to the pH 7.4 (control). Data are presented as mean ± SD (n = 3).

**FIGURE 7:**
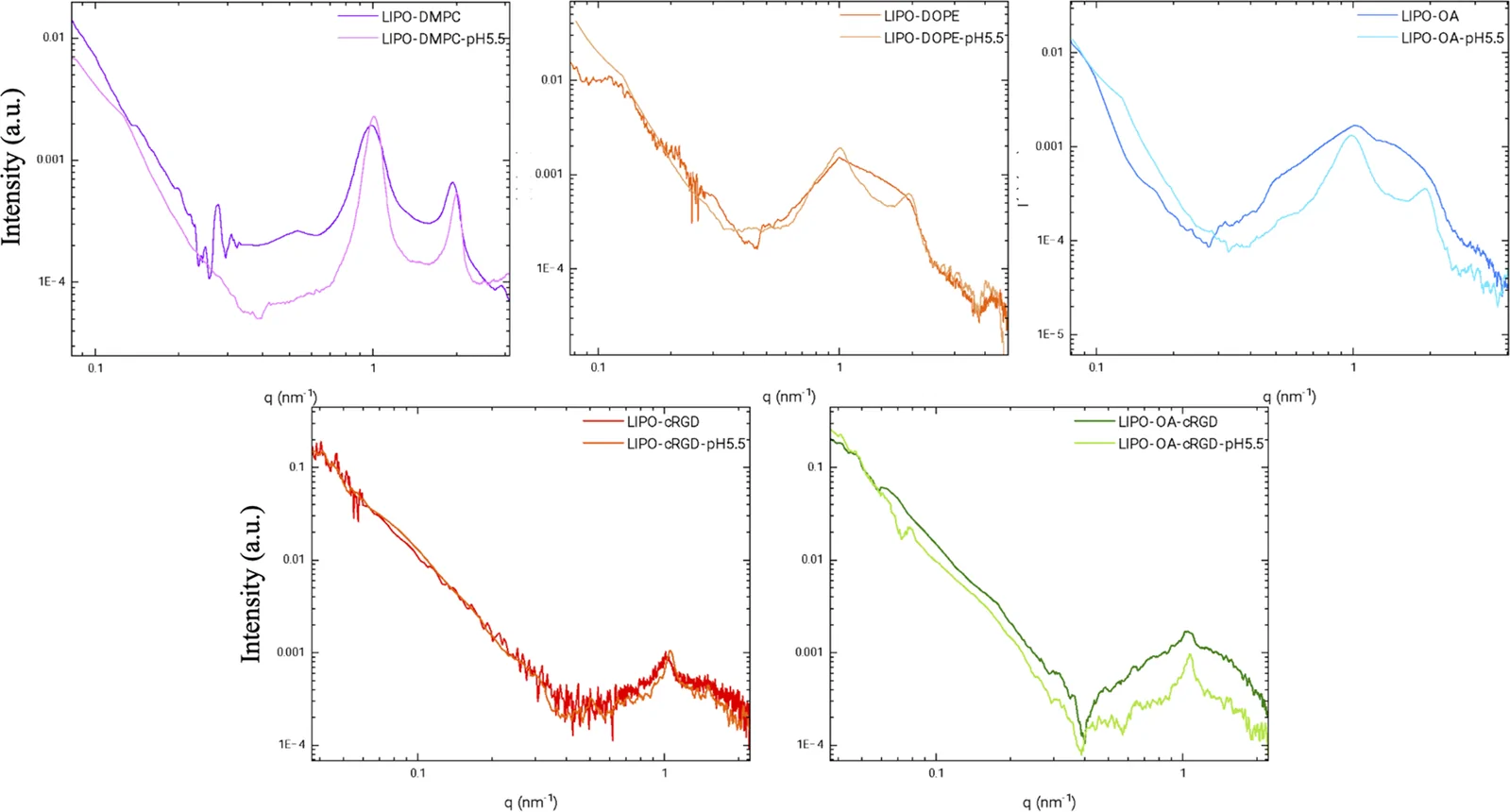
Experimental SAXS profiles for Lipo-DMPC, Lipo-DOPE, Lipo-OA, Lipo-cRGD and Lipo-OA-cRGD formulations at pH 7.4 (dark-colored curves) and pH 5.5 (light-colored lines).

To evaluate the bilayer features and its polarity, microviscosity and fluidity, two hydrophobic fluorescent probes were employed (Figure 8; Table 3). Pyrene is quite sensitive to changes in polarity of the microenvironment, also related to the different media used for sample dilution. Overall, all the polarity values ranged from 0.15 to 0.40 with no significant differences between the same samples at different pH values for Lipo-DMPC and Lipo-DOPE. In contrast, significant polarity changes upon pH reduction were observed for Lipo-OA, Lipo-cRGD, and Lipo-OA-cRGD, indicating that these formulations are more susceptible to pH-dependent membrane rearrangements. The increase in polarity observed at lower pH suggests that the probe experiences a more hydrated and polar microenvironment, likely due to changes in headgroup protonation and membrane hydration (Pentak et al., 2022). Moreover, pyrene is widely used as a fluorescent probe to evaluate membrane microviscosity because it can form excimers as a function of molecular mobility within the lipid bilayer. This parameter is particularly affected by the presence of molecules, such as OA and PEG, which are able to alter bilayer dynamics and consequently influence excimer formation (Karaz et al., 2022). Since pyrene is mainly localized in the hydrophobic inner region of the membrane, excimer formation occurs through the interaction between two pyrene molecules. In the present study, excimer formation was observed in all formulations, except for Lipo-DOPE at pH 7.4 (Figure 8B), where only a weak excimer signal was detected. This finding indicates reduced probe mobility and a lower propensity for dimer formation, suggesting that the probe is embedded in a more ordered membrane environment, in agreement with the anisotropy measurements. Consistent with these observations, Lipo-DMPC exhibited only minor spectral variations upon pH reduction, suggesting that membrane organization and probe mobility remained essentially unchanged. In contrast, DOPE-containing formulations displayed a more pronounced pH-dependent response, reflecting protonation-induced changes in lipid packing and membrane organization. Among all formulations, Lipo-OA showed the most evident increase in excimer emission at acidic pH, suggesting enhanced lateral diffusion of pyrene associated with a less tightly packed and more dynamic bilayer. Conversely, although pH-dependent changes were still observed for the PEGylated Lipo-OA-cRGD formulation, the variations in excimer intensity were less pronounced, indicating that the hydrated PEG corona partially restricts membrane reorganization and limits pyrene–pyrene encounters. Furthermore, lowering pH alters headgroup protonation and buffer–membrane interactions, affecting both local polarity (I_1_/I_3_) and probe collision frequency (excimer formation). While un-PEGylated DOPE systems (Figures 8B,C) exhibited more pronounced spectral changes upon acidification (packing and mobility changes or peptide binding), PEGylated formulations (Figures 8B1,C1) showed a dampened response, suggesting that the PEG corona increases membrane hydration while sterically shielding the lipid surface and moderating pH-induced structural rearrangements.

**FIGURE 8:**
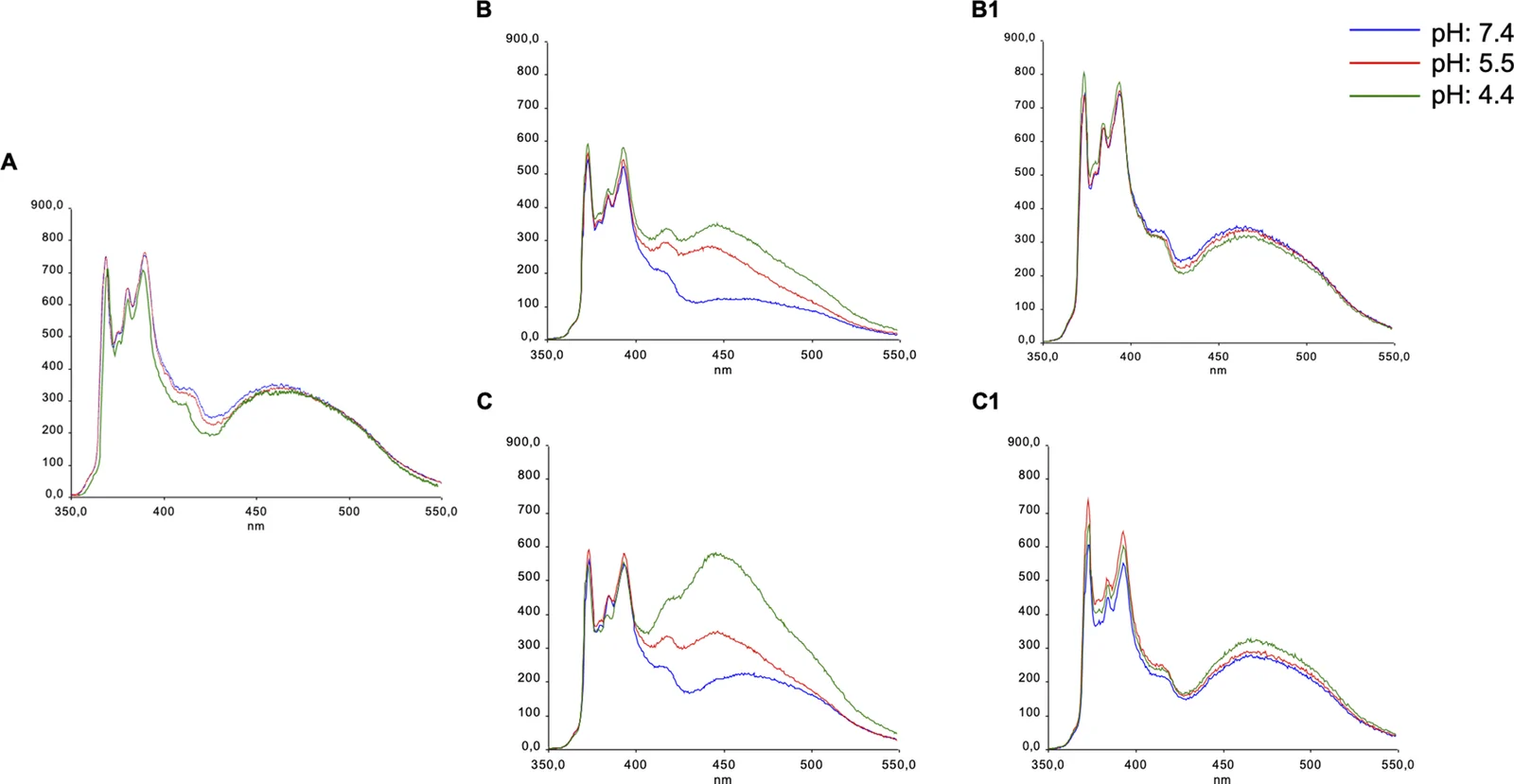
Lipo-DMPC **(A)**, Lipo-DOPE **(B)**, Lipo-OA **(C)**, Lipo-cRGD **(B1)** and Lipo-OA-cRGD **(C1)** pyrene spectra collected upon dilution with Hepes Buffer (pH 7.4, blue) and Acetate Buffer (pH 5.5, red; pH 4.4, green).

**TABLE 3:** Sample polarity, microviscosity, anisotropy values and changes of turbidity in different buffers upon acidification.

Sample	pH	Polarity (I_1_/I_3_)	Microviscosity (I_E_/I_3_)	Anisotropy (A.U.)	Turbidity (A.U.)
Lipo-DMPC	7.4	1.19	0.47	0.23	108.10
	5.5	1.17	0.47	0.22	110.11
	4.4	1.16	0.46	0.21	112.10
Lipo-DOPE	7.4	1.26	0.29	0.18	114.15
	5.5	1.28	0.64	0.15	310.56
	4.4	1.30	0.65	0.10	500.20
Lipo-OA	7.4	1.23	0.50	0.28	112.20
	5.5	1.30	0.77	0.21	456.90
	4.4	1.20	1.09	0.14	650.20
Lipo-cRGD	7.4	1.15	0.53	0.25	210.20
	5.5	1.22	0.49	0.22	301.40
	4.4	1.12	0.37	0.19	350.10
Lipo-OA-cRGD	7.4	1.34	0.63	0.29	181.45
	5.5	1.40	0.57	0.23	307.36
	4.4	1.10	0.35	0.19	380.20

The anisotropy results revealed clear pH-dependent changes in membrane organization and dynamics (Table 3). Since anisotropy values approaching 0.4 are generally associated with reduced rotational mobility of the fluorescent probe and therefore with a more rigid lipid bilayer, the lower anisotropy values observed at pH 5.5 and 4.4 indicate an overall increase in membrane fluidity upon acidification. This effect was particularly evident in the functionalized and OA-based formulations, suggesting that pH-sensitive structural rearrangements occur preferentially in membranes containing OA and/or cRGD. Among the investigated systems, Lipo-DMPC showed minimal and no significant variations in polarity, microviscosity, anisotropy, and turbidity between pH 7.4 and 4.4, indicating that the membrane remained relatively stable and structurally unaffected by acidification. Similarly, Lipo-DOPE exhibited only slight changes in anisotropy despite a marked increase in microviscosity and turbidity at acidic pH, suggesting that protonation-induced alterations mainly affected supramolecular organization and aggregation behavior rather than membrane rigidity itself.

More pronounced pH-dependent effects were observed for Lipo-OA, Lipo-cRGD, and Lipo-OA-cRGD. In particular, Lipo-OA showed a transient increase in polarity at pH 5.5 (I_1_/I_3_ from 1.23 to 1.30) followed by a decrease at pH 4.4, suggesting changes in the polarity and hydration of the bilayer interface.

At the same time, microviscosity (I_E_/I_3_) increased steadily from 0.50 to 1.09, indicating changes in the local organization of the membrane, whereas anisotropy decreased from 0.28 to 0.14, reflecting enhanced lateral mobility of the probe. Taken together, these complementary changes suggest that acidification induces substantial membrane reorganization, affecting both the local physicochemical environment and the dynamic properties of the bilayer. The presence of OA likely contributes to this behavior through protonation of its carboxylic group at acidic pH, which reduces electrostatic repulsion and destabilizes lipid packing.

The incorporation of cRGD into the liposomal membrane caused a slight increase in anisotropy at physiological pH, indicating moderate membrane stiffening. This effect can be attributed to additional hydrophobic interactions involving phospholipid acyl chains, cholesterol side chains, and the peptide-conjugated lipids, which locally restrict membrane mobility. However, upon acidification, both cRGD-functionalized formulations displayed a reduction in anisotropy, demonstrating increased membrane fluidity under acidic conditions. This behavior suggests that protonation effects weaken intermolecular interactions within the bilayer, facilitating lipid rearrangement and increasing probe rotational freedom. Interestingly, Lipo-OA-cRGD exhibited decreasing polarity values upon acidification, together with a pronounced reduction in microviscosity and anisotropy. These results suggest that extensive membrane reorganization occurs under strongly acidic conditions. The combined presence of OA and cRGD may generate a heterogeneous bilayer in which protonation-induced lipid rearrangement increases membrane fluidity while the peptide and PEG corona modulate the extent of probe mobility and membrane destabilization. Finally, all formulations except Lipo-DMPC exhibited a marked increase in turbidity upon acidification, strongly suggesting the occurrence of pH-dependent interactions. This effect was especially pronounced in Lipo-OA and Lipo-OA-cRGD, supporting the hypothesis that protonation-induced changes in membrane surface charge and lipid packing reduce colloidal stability and promote intervesicular interactions (Moffa et al., 2026). Overall, these findings demonstrate that acidic pH significantly affects membrane dynamics, particularly in OA- and cRGD-containing formulations, leading to increased fluidity, altered microenvironment polarity, and enhanced aggregation propensity related to increased turbidity.

The rhodamine-based assay provided clear evidence of the pH-sensitive nature of the cRGD-functionalized liposomal systems. Specifically, under physiological conditions (pH 7.4), the liposomal samples remain largely colorless and non-fluorescent. Upon acidification (pH ranging from 4.4 to 5.5), protonation induces destabilization of the lipid bilayer, leading to the appearance of a pink color and the onset of fluorescence (Figures 9A,B). This effect was particularly pronounced in OA-based cRGD-functionalized and PEGylated liposomes, highlighting the role of lipid composition and surface modification in modulating pH responsiveness.

**FIGURE 9:**
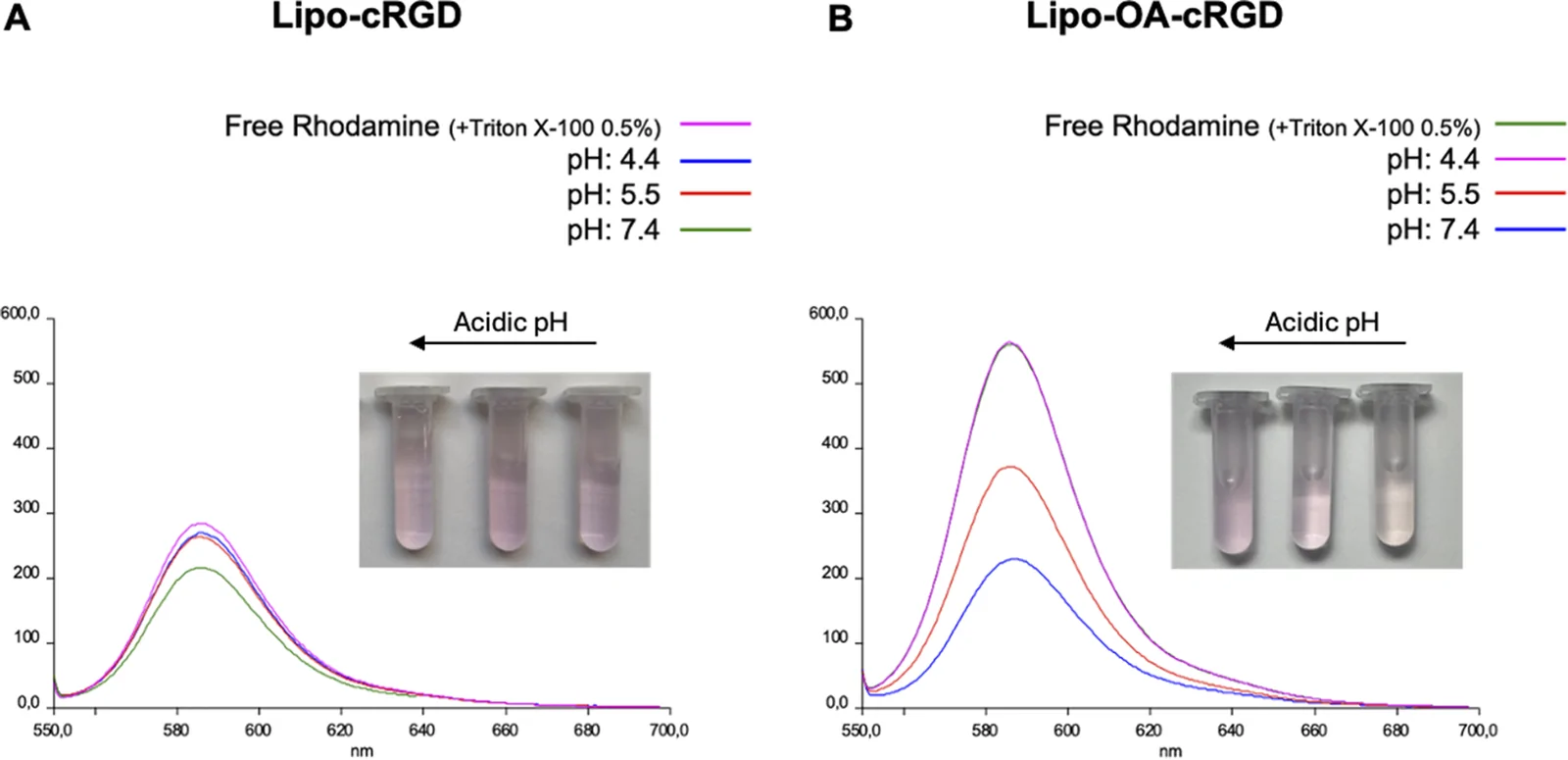
Fluorescence emission spectra of Rhodamine-loaded Lipo-cRGD **(A)** and Lipo-OA-cRGD **(B)** recorded under different pH conditions and representative photographs of liposomal suspensions at different pH values showing the progressive color change from colorless to pink upon acidification, indicative of pH-triggered rhodamine release.

The release studies demonstrated the release behavior of FITC and Calcein from different liposomal formulations at physiological (pH 7.4) and acidic pH (5.5 and 4.4). Free fluorescent probes rapidly diffused through the dialysis membrane, reaching complete release within 1–2 h regardless of the pH conditions. As expected, the non-pH-responsive DMPC liposomes showed comparable release profiles at all tested pH values. FITC release gradually increased over time, reaching approximately 45%–50% after 72 h with no statistically relevant differences between pH 7.4, 5.5, and 4.4 (Figure 10A). Similarly, Calcein-loaded DMPC liposomes exhibited only a modest increase in cumulative release, remaining below approximately 40% after 72 h irrespective of the external pH (Figure 10A1). These results confirm that DMPC liposomes maintain a relatively stable membrane permeability over the investigated pH range and therefore serve as an appropriate non-pH-sensitive control. In contrast, liposomal formulations containing DOPE or OA showed a release profile strongly dependent on both membrane composition and environmental pH. At physiological pH (7.4), Lipo-DOPE liposomes exhibited lower cargo release (approximately 50% of FITC after 72 h), with respect to OA-containing liposomes (Lipo-OA), displaying faster cargo release under neutral conditions (about 60% within 48 h) (Figure 10B). When the pH was reduced to 5.5, mimicking the mildly acidic tumor microenvironment and endosomal compartments, both pH-sensitive formulations exhibited a pronounced increase in FITC release. Lipo-DOPE released approximately 75%–80% of the encapsulated FITC after 72 h, while Lipo-OA reached nearly complete release (≈95–100%). A further decrease to pH 4.4, representative of the late endosomal/lysosomal environment, enhanced this behavior even more, with both formulations approaching complete FITC release, confirming the preservation of their pH-responsive membrane destabilization under increasingly acidic conditions (Figure 10B).

**FIGURE 10:**
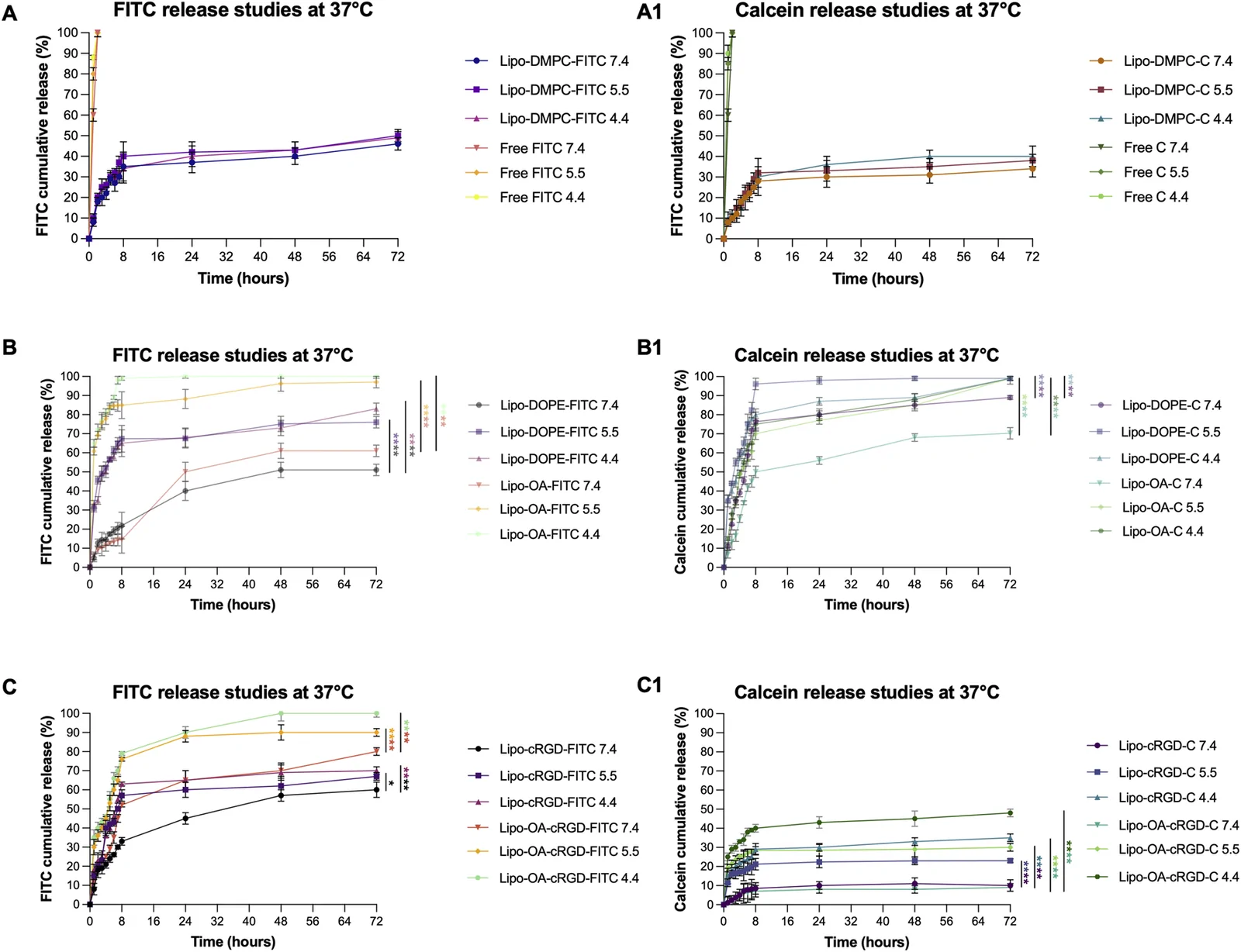
Release profile of FITC- **(A–C)** and Calcein-loaded liposomes **(A1–C1)** at physiological pH (7.4) and acidic pH (5.5 and 4.4). Free FITC and **(C)** releases are plot in graph **(A,A1)**, respectively. Data are presented as mean ± SD (n = 3). Statistical significance is indicated by asterisks (*p < 0.05, **p < 0.01, ***p < 0.001, ****p < 0.0001). For formatting purposes, only p-values <0.05 are shown. Statistical analysis was performed for pH 7.4 *vs*. pH 5.5 and pH 7.4 *vs*. pH 4.4.

A similar trend was observed for Calcein-loaded formulations (Figure 10B1). The non-PEGylated liposomes exhibited substantial Calcein leakage, particularly under acidic conditions. At pH 7.4, Lipo-DOPE released approximately 90% of Calcein after 72 h, whereas Lipo-OA retained a greater fraction of the probe, reaching about 70% release. Acidification markedly accelerated membrane permeabilization, with both formulations approaching complete release at pH 5.5 and 4.4. The higher Calcein release observed from DOPE-containing liposomes at pH 7.4 further highlights that pH responsiveness does not imply complete cargo retention under physiological conditions.

Figure 10C and Figure 10C1 further demonstrate that cRGD-functionalized liposomes preserved the pH-responsive behavior of the parent formulations while exhibiting improved cargo retention. For FITC-loaded liposomes (Figure 10C), both cRGD-DOPE and cRGD-OA formulations displayed a clear pH-dependent release profile. At physiological pH (7.4), FITC release remained relatively limited, reaching approximately 60% and 75% after 72 h for Lipo-cRGD and Lipo-OA-cRGD, respectively. Upon acidification to pH 5.5, the cumulative release significantly increased, approaching approximately 90% for the OA-containing formulation and about 70% for Lipo-cRGD. An even more pronounced release was observed at pH 4.4, where Lipo-OA-cRGD reached nearly complete release, while Lipo-cRGD exhibited a moderate but significant increase compared with physiological conditions. These findings indicate that cRGD conjugation does not impair the acid-triggered destabilization of the lipid bilayer, although it partially moderates the release kinetics compared with the corresponding non-functionalized liposomes.

A more evident effect of cRGD functionalization was observed for Calcein-loaded liposomes (Figure 10C1). Since Calcein is highly hydrophilic, its retention strongly depends on the integrity and packing density of the lipid bilayer. Protonation of lipid headgroups at acidic pH likely destabilizes membrane packing, increases bilayer permeability, and facilitates diffusion of Calcein into the external medium. Accordingly, in contrast to the non-PEGylated formulations (Figure 10B1), cumulative Calcein release remained substantially lower under all experimental conditions. At pH 7.4, both formulations released less than approximately 10% of the encapsulated probe after 72 h, indicating excellent retention under physiological conditions. Acidification to pH 5.5 and, more markedly, to pH 4.4 increased membrane permeability, leading to higher cumulative release. Nevertheless, Calcein release remained below approximately 35% for Lipo-cRGD and below 50% for Lipo-OA-cRGD, even under the most acidic conditions. The lower leakage compared with the non-functionalized liposomes is likely attributable to the PEG spacer used for cRGD conjugation. PEG chains generate a steric barrier at the liposome surface, which stabilizes the membrane and limits water penetration (Nag and Awasthi, 2013). This steric shielding decreases membrane permeability and therefore restricts the escape of hydrophilic molecules such as Calcein (Makharadze et al., 2025). The dense hydrated PEG corona likely acts as an additional diffusion barrier for Calcein, slowing its outward transport even when membrane destabilization occurs (Nguyen et al., 2022). Consequently, although acidic pH still promotes membrane destabilization and payload release, PEGylation effectively provide steric stabilization, reducing membrane permeability and improving cargo retention, while preserving the desired pH-responsive behavior. Overall, the pH-sensitivity assessments consistently demonstrated that the multi-technique characterization successfully confirmed the preservation of the pH-responsive behavior of the liposomal formulations after cRGD functionalization, in contrast to the non-pH-sensitive DMPC control. Although cRGD conjugation partially reduced the overall release efficiency, particularly for the hydrophilic probe, the formulations retained a clear pH-triggered release profile. Specifically, the maximum release at acidic pH reached approximately 40% for the hydrophilic probe, whereas FITC release remained markedly higher, reaching about 80%–90% under the same conditions. Finally, the designed systems provide a proof of concept for pH-responsive drug delivery, supporting their potential for controlled payload release under acidic conditions.

Taken together, the results provide complementary evidence that the developed liposomal system retains its pH-sensitive properties following cRGD functionalization, while simultaneously acquiring targeting functionality (Figure 11).

**FIGURE 11:**
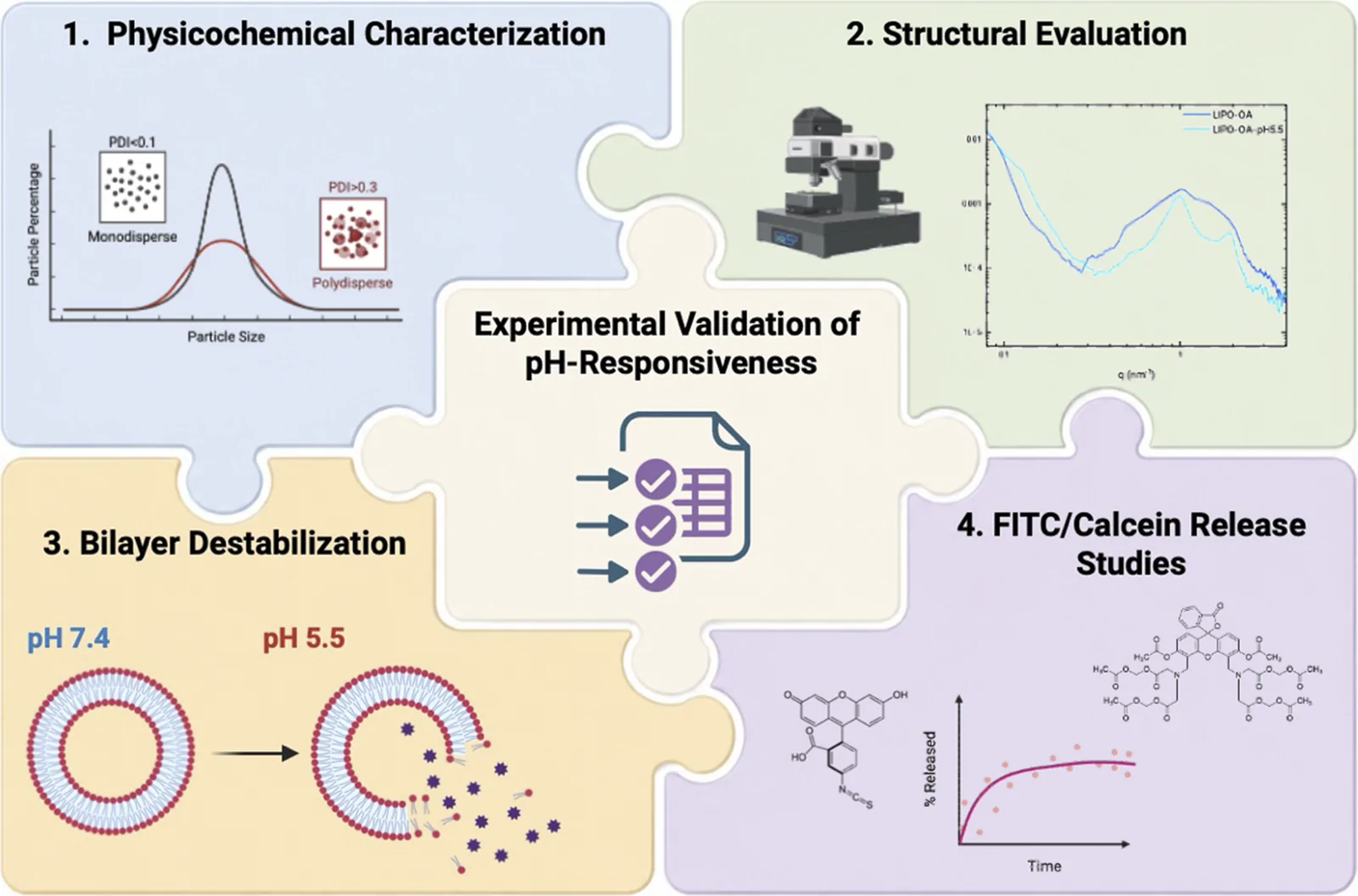
Conceptual illustration showing how complementary experimental findings collectively validate the pH-responsive behavior of the developed liposomal platform. Each experimental approach contributes a key piece of evidence toward confirming the structural and functional response of the liposomes to acidic pH.

### 3.4 Stability studies over time

The physicochemical stability over time of the liposomal formulations was evaluated by monitoring H_D_ and ζ-potential over time under different storage conditions. At both room temperature RT and 4 °C, all formulations maintained relatively stable size distributions and surface charge values throughout the experimental period, indicating good colloidal stability (Figures 12, 13). In particular, for cRGD-coated liposomes (Figure 13), the H_D_ remained within a narrow dimensional range, generally between 100 and 200 nm, under all tested conditions even if at both RT and 4 °C only minor fluctuations in particle size and ζ-potential were observed, confirming overall the preservation of vesicle integrity during storage. The formulations stored at 4 °C exhibited slightly lower variability compared to RT, suggesting improved stability at lower temperature.

**FIGURE 12:**
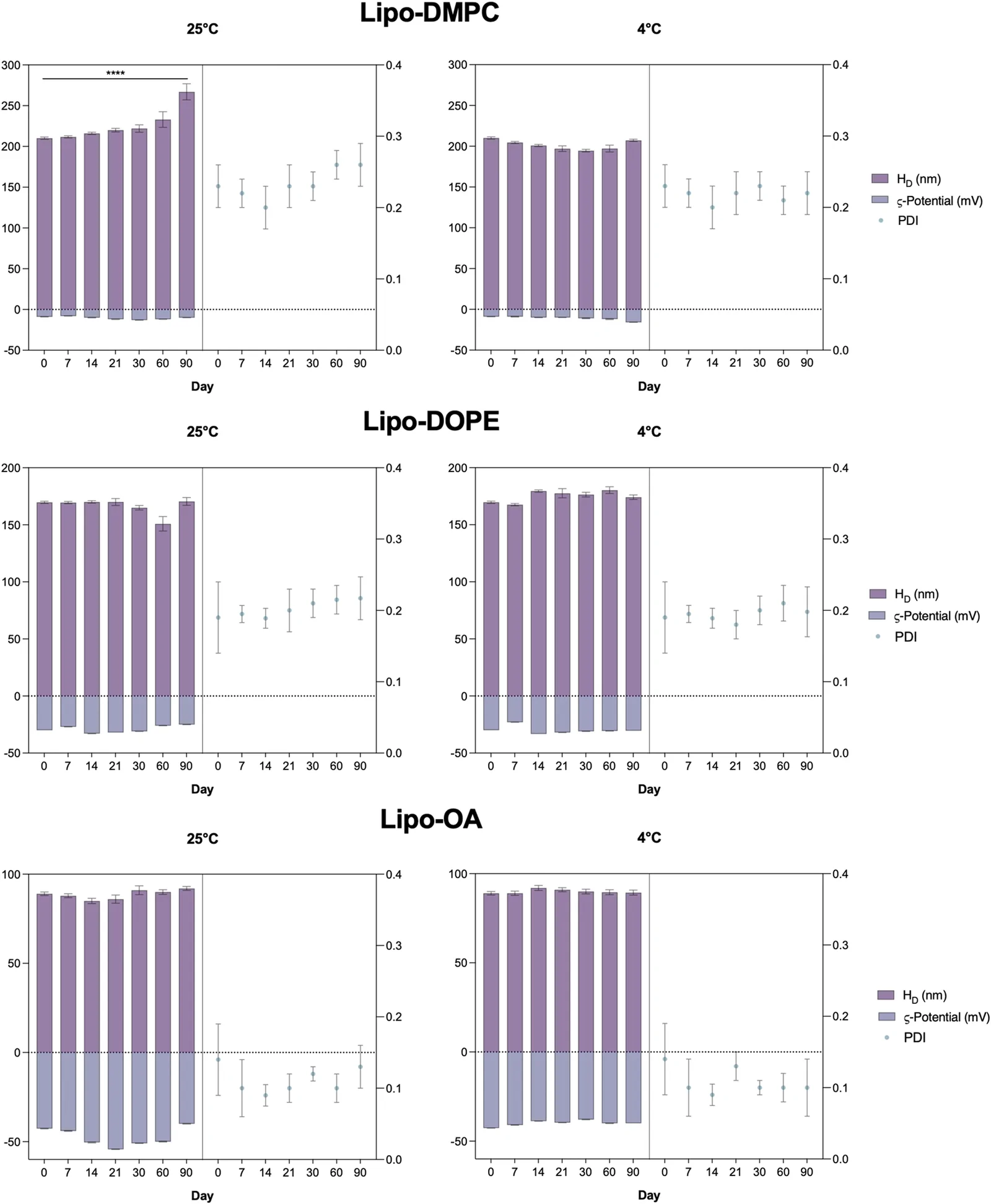
Stability studies of liposomal formulations stored at room temperature (RT) and 4 °C over a period of 90 days. H_D,_ PDI, and ζ-potential were monitored over time to evaluate the long-term physicochemical stability of the formulations under different storage conditions.

**FIGURE 13:**
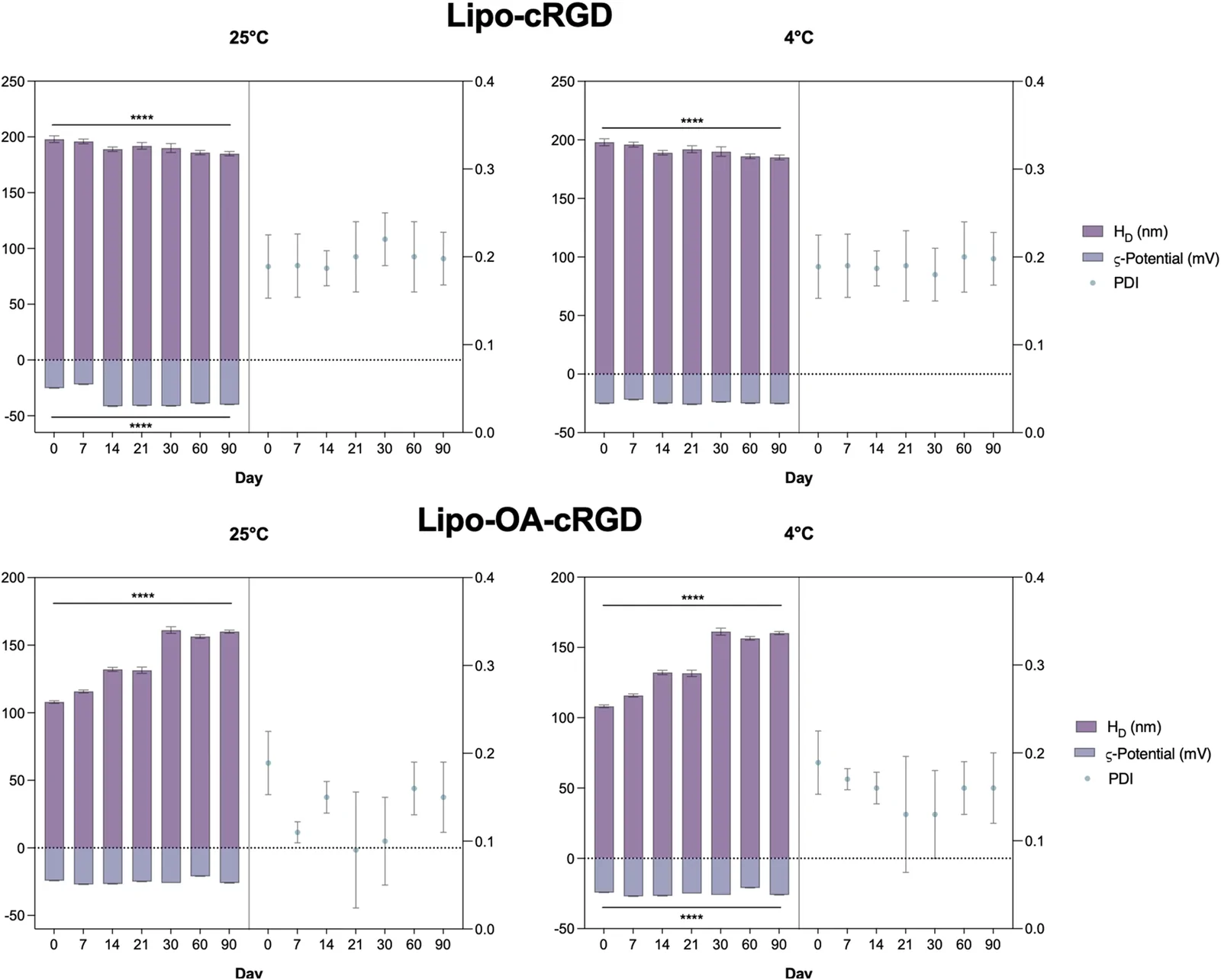
Stability studies of cRGD-coated liposomal formulations stored at room temperature (RT) and 4 °C over a period of 90 days. H_D,_ PDI, and ζ-potential were monitored over time to evaluate the long-term physicochemical stability of the formulations under different storage conditions.

To preliminarily assess the biological stability of the developed liposomal formulations under physiologically relevant conditions, serum stability studies were performed in HS at 37 °C (Figure 14). Evaluation of nanoparticle behavior in serum-containing environments is essential because interactions with proteins and other serum components can strongly influence colloidal stability, membrane integrity, surface properties, and ultimately the *in vivo* performance of liposomal systems (Abstiens et al., 2019). In particular, serum proteins may adsorb onto the liposomal surface, leading to the formation of a protein corona that can alter particle size, aggregation tendency, and circulation behavior. For this reason, changes in hydrodynamic diameter and colloidal stability were monitored over time in order to investigate the ability of the formulations to maintain their physicochemical properties under conditions that mimic the systemic biological environment.

**FIGURE 14:**
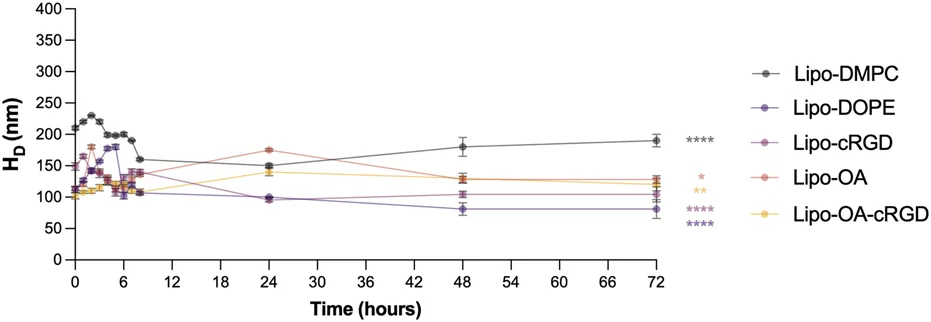
Liposomal stability studies carried out in HS up to 72 h at 37 °C.

Interestingly, all formulations exhibited a transient increase in diameter at intermediate time points (before 6 h), followed by progressive stabilization during prolonged incubation up to 72 h. This behavior may reflect an initial dynamic interaction between serum proteins and the liposomal surface, followed by equilibration of the protein corona and stabilization of the nanoparticle interface. Among the tested systems, cRGD-based systems displayed the most stable size profile over time, suggesting that surface functionalization with cRGD contributes to improved colloidal stability under physiological conditions (Santhanakrishnan et al., 2024). The presence of PEGylated phospholipids and peptide moieties may provide steric stabilization effects that limit extensive interparticle interactions and reduce serum-induced aggregation (Folda et al., 2025). Importantly, although some variations in hydrodynamic diameter were found to be statistically significant, all formulations maintained particle sizes within the expected nanoscale range throughout the incubation period. Moreover, count rate measurements did not reveal substantial decreases or abnormal fluctuations, indicating that the liposomal suspensions remained colloidally stable and that no major disruption, precipitation, or extensive aggregation phenomena occurred (Vogel et al., 2021). These findings further support the preservation of vesicle integrity under serum conditions. Overall, the stability observed in HS suggests that the lipid bilayers remain sufficiently intact to preserve vesicle structure and potentially retain the encapsulated cargo under physiological conditions. Collectively, these findings demonstrate that all liposomal formulations exhibit adequate serum compatibility and stability at physiological temperature, supporting their potential suitability for subsequent *in vitro* and *in vivo* biological applications.

Calcein and FITC release profiles were evaluated under physiologically relevant conditions to assess the stability and retention properties of the liposomal systems. Fluorescence measurements were performed at predetermined time points to monitor the progressive release of encapsulated calcein and FITC. As shown in Figure 15, all the samples exhibited a time-dependent release behavior, also confirming their ability to maintain vesicle integrity during prolonged incubation in serum-containing media. All the formulations exhibited a rapid initial release phase during the first 8 h of incubation, followed by a slower and more sustained release pattern at longer time points. This biphasic behavior is characteristic of liposomal systems and may reflect the initial release of surface-associated or weakly entrapped molecules, followed by the gradual diffusion of the encapsulated probes through the lipid bilayer (Kandregula et al., 2022).

**FIGURE 15:**
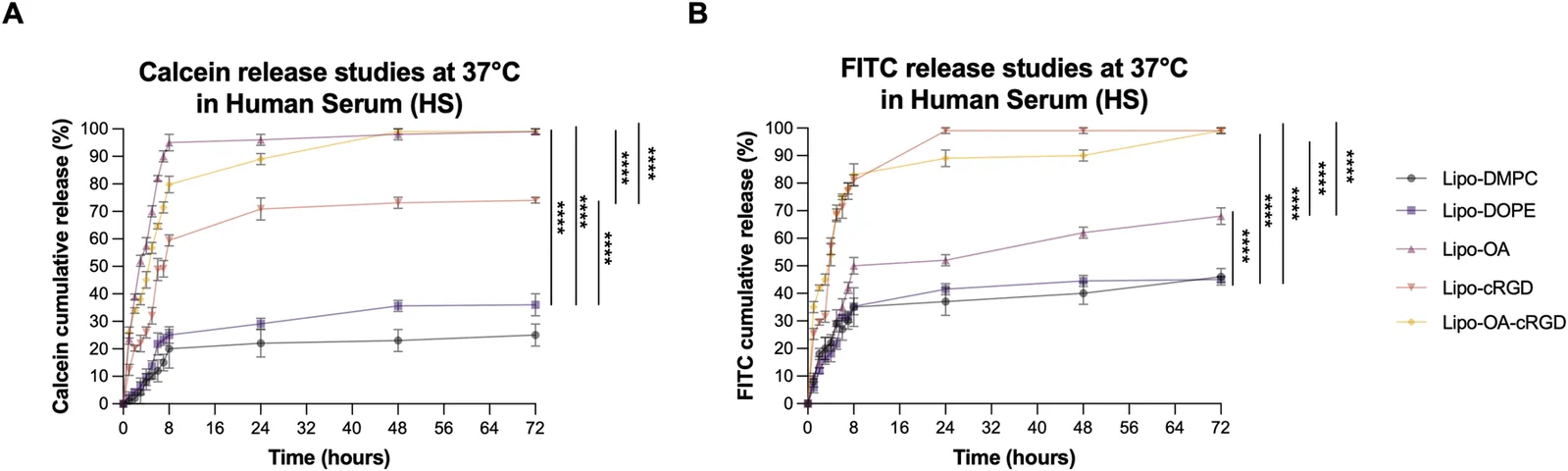
*In vitro* release studies of calcein‐ **(A)** and FITC‐loaded **(B)** liposomal formulations in HS at 37 °C over 72 h.

Among all formulations, Lipo-DMPC exhibited the highest membrane stability under human serum conditions. Calcein release remained below approximately 25% after 72 h, while FITC release reached only about 45%, indicating limited membrane permeability and efficient retention of both hydrophilic and amphiphilic probes. The absence of pH-responsive lipids and surface modifications likely contributes to the compact organization of the DMPC bilayer, making this formulation an appropriate reference for evaluating the effects of DOPE, OA, and cRGD functionalization on serum stability.

For both calcein and FITC, Lipo-DOPE displayed a slow-release kinetics, reaching approximately 35%–45% cumulative release after 72 h. This behavior suggests a relatively stable and compact membrane organization capable of efficiently retaining the encapsulated fluorophores under serum conditions. In contrast, cRGD-functionalized formulations exhibited significantly faster release profiles. In particular, Lipo-cRGD showed an almost complete release of calcein within the first 24–48 h, indicating enhanced membrane permeability and/or reduced bilayer packing stability induced by peptide functionalization in HS. The incorporation of OA also markedly influenced release behavior. Lipo-OA exhibited intermediate release kinetics, with cumulative release values approaching 70%–75% after prolonged incubation. This effect is likely related to the ability of OA to perturb lipid packing, thereby facilitating diffusion of the encapsulated compounds. Interestingly, the combined presence of OA and cRGD in Lipo-OA-cRGD resulted in the fastest and most extensive release profiles, particularly for FITC, where nearly complete release was observed within 24 h. These findings suggest a synergistic contribution of OA-induced membrane destabilization and cRGD-mediated structural perturbation in promoting serum-triggered leakage. Notably, the release behavior was comparable for both fluorescent probes, although FITC-loaded formulations generally exhibited slightly faster release kinetics than calcein-loaded systems. This difference may be associated with the distinct physicochemical properties of the fluorophores, including molecular size, hydrophilicity, and interactions with the lipid bilayer. Overall, the obtained results demonstrate that lipid composition and surface functionalization strongly influence release kinetics under biologically relevant conditions. Although all formulations maintained their colloidal stability during incubation in HS, differences in lipid composition markedly affected the release kinetics of both fluorescent probes. OA- and cRGD-containing formulations exhibited higher membrane permeability and consequently faster probe release than DMPC- and DOPE-based liposomes, highlighting the importance of optimizing membrane composition to balance serum stability with efficient payload release.

## 4 Conclusion

In this study, pH-sensitive liposomal formulations based on DOPE, OA, and cRGD functionalization were successfully produced using a microfluidic approach, yielding nanosized vesicles with low polydispersity and suitable colloidal properties for drug delivery applications. Physicochemical characterization demonstrated that all formulations exhibited hydrodynamic diameters within the optimal range for nanocarrier applications and adequate surface charge to ensure colloidal stability. The pH-responsive behavior of the formulations was consistently demonstrated through the combined application of multiple complementary techniques (as previously shown in Figure 11). This behavior was absent in the non-pH-sensitive Lipo-DMPC formulation, confirming the key role of lipid composition in mediating acid-triggered structural transitions. Fluorescence spectroscopy and membrane dynamics analyses further demonstrated that acidic pH induces significant alterations in bilayer organization, particularly in OA-containing and cRGD-functionalized liposomes. These changes included increased membrane hydration, modified polarity, enhanced fluidity, and altered microviscosity, all indicative of protonation-driven membrane rearrangements. The incorporation of OA contributed to pH-triggered destabilization, while cRGD functionalization modulated membrane organization and responsiveness without compromising the pH-sensitive behavior of the systems. Overall, the results highlight the potential of OA- and cRGD-containing liposomes as smart nanocarriers capable of undergoing structural and physicochemical transitions under mildly acidic conditions, such as those encountered in endosomal compartments. These properties may enhance intracellular delivery and support the future development of targeted and stimuli-responsive liposomal systems for therapeutic applications.

## Data Availability

The original contributions presented in the study are included in the article/Supplementary Material, further inquiries can be directed to the corresponding author.
