# Supplementary material for: From conventional to pH-sensitive formulations: increasing complexity, increasing characterization
Source: Front Bioeng Biotechnol. 2026 Oct 28;14:1999697. doi: 10.3389/fbioe.2026.1999697 (PMC13752915; doi:10.3389/fbioe.2026.1999697)
Supplement: Supplementary file 1 [file DataSheet1.docx]

Supplementary Material

# 1 Supplementary Figures and Tables

## 1.1 Supplementary Figures

**Supplementary Figure 1.** The structural evolution of Lipo-DMPC (A), Lipo-DOPE (B) and Lipo-OA (C) formulations under acidic pH were assessed by comparing SAXS scattered intensity profiles at pH 7.4 and 5.5. The intensity profiles were analysed using SasView software (version 6.0.0), using a random lamellar head/tail/tail/head sheet with Caillé structure factor model, suitable for the description of stacked lipid bilayers. The overall membrane thickness, the lamellar repeat distance, the number of correlated layers, and bilayer fluctuations through the Caillé parameter could be obtained (**Supplementary Table 1**). The fitted parameters were used to compare the degree of lamellar ordering among the different liposomal formulations.

**Supplementary Figure 2.** The structural evolution of Lipo-cRGD (A) and Lipo-OA-cRGD (B) formulations at pH 7.4 and 5.5. The intensity profiles were analysed using SasView software (version 6.0.0), using a random lamellar head/tail/tail/head sheet (**Supplementary** **Table 2**).

## 1.2 Supplementary Tables

**Supplementary Table 1.** Data related to Figure 1S.

|  | LIPO-DMPC | LIPO-DMPC  pH 5.5 | LIPO-DOPE | LIPO-DOPE  pH 5.5 | LIPO-OA | LIPO-OA  pH 5.5 |
| --- | --- | --- | --- | --- | --- | --- |
| Bilayer | 39 | 47 | 47 | 38 | 46 | 44 |
| Head | 9.3 | 8 | 11 | 8 | 11 | 10 |
| Tail | 10.2 | 15.6 | 12.5 | 11 | 12 | 12 |
| Sld_head | 10.9 | 10.3 | 12.5 | 12.6 | 12 | 11.1 |
| Sld_tail | 7.6 | 7.23 | 7 | 6.2 | 7.15 | 8 |
| D_spacing | 63.7 | 62.5 | - | 62.9 | - | 64 |
| Caille | 0.17 | 0.09 | - | 0.3 | - | 0.25 |

**Supplementary Table 2.** Data related to Figure 2S.

|  | LIPO-cRGD | LIPO-cRGD pH 5.5 | LIPO-OA-cRGD | LIPO-OA-cRGD pH 5.5 |
| --- | --- | --- | --- | --- |
| Head | 8 | 8 | 11.5 | 13 |
| Tail | 13.3 | 13.3 | 13 | 13 |
| Sld_head | 11.7 | 11.7 | 11.14 | 10.3 |
| Sld_tail | 8 | 8 | 7.98 | 8.5 |
